# Activin/follistatin system in grass carp pituitary cells: - Regulation by local release of growth hormone and luteinizing hormone and its functional role in growth hormone synthesis and secretion

**DOI:** 10.1371/journal.pone.0179789

**Published:** 2017-06-29

**Authors:** Roger S. K. Fung, Jin Bai, Karen W. Y. Yuen, Anderson O. L. Wong

**Affiliations:** School of Biological Sciences, the University of Hong Kong, Pokfulam Road, Hong Kong, China; University of Macau, MACAO

## Abstract

Gonadotrophin regulation by activin/follistatin system is well-documented, but the corresponding effect on growth hormone (GH) has not been fully characterized and with little information available in lower vertebrates, especially in fish models. In grass carp, local interactions of GH and luteinizing hormone (LH) can induce GH release and gene expression at pituitary level via autocrine/paracrine mechanisms. To shed light on the role of activin/follistatin system in GH regulation by local actions of GH and LH, grass carp activin βA and βB were cloned, shown to be single-copy genes expressed in the pituitary, and confirmed to encode activin proteins capable of transactivating promoter with activin-responsive elements. In grass carp pituitary cells, activin A and B were effective in reducing GH secretion and GH cell content with concurrent drop in GH mRNA level whereas the opposite was true for follistatin, the activin-binding protein known to neutralize the effects of endogenous activin. Treatment with activin A and B not only could suppress basal but also inhibit GH mRNA expression induced by GH and human chorionic gonadotropin (hCG), a functional analogue of LH in fish model. Apparently, down-regulation of GH mRNA by activin was mediated by reducing GH transcript stability with concurrent inhibition on GH promoter activity via the SMAD pathway. In reciprocal experiments, GH treatment was found to up-regulate activin βA, activin βB and follistatin mRNA levels in carp pituitary cells but the opposite was noted by removing endogenous GH with GH antiserum. Interestingly, parallel treatment with hCG could also inhibit basal as well as GH-induced activin βA, activin βB and follistatin gene expression. These results, as a whole, indicate that the pituitary activin/follistatin system can serve as a regulatory target for local interactions of GH and LH and contribute to GH regulation by autocrine/paracrine mechanisms in the carp pituitary.

## Introduction

Activin, a member of TGFβ superfamily, was first isolated from follicular fluid by its stimulatory action on follicle-stimulating hormone (FSH) release in pituitary cells [1, 2] It is a dimeric protein composed of two β subunits, which are known to have two major forms, namely βA and βB subunits. Different combination of the two isoforms of β subunits can lead to the formation of activin homodimer (activin A with βA:βA subunits or activin B with βB:βB subunits) or heterodimer (activin AB with βA:βB subunits) [3]. Activin is widely expressed at tissue level and serves an autocrine/paracrine factor with diverse functions including folliculogenesis, spermatogenesis, production of sex steroids, local regulation of pituitary hormones, stem cell survival and embryo development, cell proliferation and differentiation, wound healing/tissue repairing and immune responses [4–10]. The biological effects of activin are mediated via type I (ActRI) and type II activin receptors (ActRII) [11], and to a less extent by type II BMP receptor [12]. These receptors are members of serine/threonine kinase receptor family functionally coupled to the SMAD pathway, especially with SMAD2 and SMAD3 for activin signalling [11, 13]. In cancer patients, aberrant signalling via activin and/or its receptors can be linked with cancer progression including epithelial-to-mesenchymal transition, cancer cell migration and invasion and neo-angiogenesis during tumorigenesis [14].

In mammals, the reproductive function of activin is well-documented. Its involvement in oocyte recruitment and maturation has been extensively studied [8] and shown to be well conserved in lower vertebrates including fish models [15]. The regulatory effects of activin on reproductive functions can also exert via autocrine/paracrine actions within the pituitary. In rodents (e.g., rat), activin is expressed mainly in gonadotrophs [16] and stimulates FSH secretion and gene expression with concurrent up-regulation of GnRH receptor [17]. The stimulatory effect of activin on FSH gene expression is mediated by the synergistic action of SMADs and FoxL2 acting on the proximal region of FSH promoter [18, 19]. At pituitary level, FSH induction by activin is subjected to the inhibition by inhibin from the gonad and local production of follistatin [4, 17, 20]. Pituitary expression of follistatin, the binding protein for activin, can be up-regulated by activin to form a local feedback to suppress the FSH responses, probably by preventing activin from binding to its receptor on gonadotrophs [21]. In general, activin is considered to be a FSH stimulator with little/no effect on luteinizing hormone (LH), but activin-induced LHβ gene expression has been reported in some studies, e.g., in LβT2 cells [22]. Besides gonadotropins, activin is also known to have regulatory actions on other pituitary hormones but the results published are highly variable among different research groups. For examples, in primary culture of pituitary cells or pituitary adenomas/cell lines (e.g., GH3 cells), both stimulatory [23, 24] and inhibitory effects of activin [25–27] on growth hormone (GH) release and gene expression have been reported. The cause of the discrepancy is unclear and the biological relevance of these findings still remains to be elucidated.

In fish models, the pituitary action of activin on gonadotropin regulation appears to be similar and yet distinct from that of mammals. At the pituitary level, activin was shown to have differential effects on LH and FSH expression, with stimulation on FSHβ but inhibition on LHβ gene expression, e.g., in zebrafish [28], goldfish [29] and more recently in European eel [30]. In goldfish, pituitary expression of follistatin has been confirmed [31] and the differential effects on LH and FSH by activin could be blocked/reverted by co-treatment with follistatin [32], suggesting the presence of an activin/follistatin system in the fish pituitary similar to that of mammals. Despite the inhibitory effect of activin on LHβ gene expression, activin and its functional antagonist inhibin were both effective in inducing LH release in goldfish pituitary fragments [33]. For GH regulation, activin was found to be expressed in goldfish somatotrophs and stimulate GH secretion in goldfish pituitary cells, presumably via autocrine/paracrine mechanisms [34]. In zebrafish pituitary cells, GH mRNA expression could be attenuated by activin but similar treatment with follistatin was without effects [28]. However, a recent study in European eel has shown that activin treatment did not alter GH gene expression at pituitary level [30], suggesting that the GH responses to activin may be species-specific in fish models.

In grass carp, a cultured species of Cyprinids with high commercial value in Asian countries, a zonal distribution of pituitary cells can be noted in the pituitary with gonadotrophs and somatotrophs present in close proximity to each other [35], which provides the anatomical basis for local interactions between the two cells types. In grass carp pituitary cells, autoregulation of GH [36] as well as other members of GH gene lineage including prolactin (PRL, [37]) and somatolactin [38] have been reported. In the carp pituitary, local release of GH was shown to induce PRL release and gene expression and these paracrine effects of GH could be inhibited by local release of LH [37]. In the same animal model, an intrapituitary feedback loop for GH regulation via autocrine/paracrine interaction of goandotrophs and somatotrophs has been reported [39]. In this feedback loop, LH release from goanatotrophs can induce GH secretion and gene expression in somatotrophs via paracrine actions [40]. At the level of somatotrophs, the effects of LH can be further enhanced by autocrine amplification via GH-induced GH gene expression [36] and GH released locally in turn can exert a negative feedback to suppress LH secretion in neighbouring gonadotrophs [41]. These findings, as a whole, suggest that local interaction of pituitary hormones may constitute a new facet for pituitary regulation in fish model.

Although the activn/follistatin system has been confirmed to be an autocrine/paracrine component for pituitary regulation in fish model, its functional role in local interaction between gonadotrophs and somatotrophs has not been examined. In this study, using grass carp as a model, we test the hypothesis that the activin/follistatin system may serve as a regulatory target for GH and LH released locally at the pituitary level and contribute to the autocrine/paracrine regulation of GH synthesis and secretion in carp species. As a first step, grass carp activin βA and βB were cloned, their structural features, gene copy number and tissue expression pattern were characterized, and the bioactivity of protein products encoded by the two activin β subunits was confirmed by functional expression. Using grass carp pituitary cells and GH3 somatotroph cells as cell models, the effects of activin A and B as well as follistatin treatment on GH secretion and GH cell content were examined and correlated with the respective changes in GH mRNA expression. The possible involvement of transcriptional regulation of GH promoter activity and post-transcriptional modification of GH transcript stability were also tested for GH regulation by activin. In reciprocal studies with carp pituitary cells, the effects of GH treatment on activin βA, activin βB and follistatin mRNA expression were also investigated and the functional role of endogenous GH released in pituitary cell culture was also confirmed by immunoneutralization with GH antiserum. To shed light on the role of LH secretion in activin/follistatin regulation, parallel studies were conducted with human chorionic gonadotropin (hCG), a functional analogue of LH in fish models. In this case, both basal as well as the effects of GH on activin βA, activin βB and follistatin gene expression were examined with hCG treatment and the functional role of endogenous LH released in pituitary cells was also tested by immunoneutralization with antiserum raised against carp LH. Our studies, as a whole, provide evidence that the activin/follistatin system is involved in GH regulation in grass carp and pituitary expression of activin and follistatin can be modified by functional interactions of GH and LH released locally in the carp pituitary. These findings suggest that the activin/follistatin system may be as a new component of the intrapituitary feedback loop for GH regulation mediated by local interactions of gonadotrophs and somatotrophs in carp species.

## Materials and methods

### Animals and test substances

Mixed sexes of one-year-old grass carp (*Ctenopharyngodon idellus*) with body weight of 1.8–2.3 kg were obtained from Western wholesale market (Shek Tong Tsui, Hong Kong) and maintained in well-aerated aquaria at 20°C under a 12-hr light:12-hr dark photoperiod for two weeks prior to experimentation. During the process of tissue sampling or pituitary cell preparation, the fish were sacrificed by anaesthesia in 0.05% MS222 (Sigma-Aldrich, St. Louis, MO) followed by spinosectomy according to the protocol (CULATR No.3890) approved for this study by the Committee for Animal Use in Teaching and Research at the University of Hong Kong (Hong Kong). For drug treatment in cell culture, porcine GH, hCG, equine LH and equine FSH were obtained from Sigma-Aldrich and human activin A, activin B and follistatin as well as the pharmacological inhibitors targeting different pathways including (*E*)-2-cyano-3-(3,4-dihydrophenyl)-*N*-(phenylmethyl)-2-propenamide (AG490), N1-(11H-indolo[3,2-c]quinolin-6-yl)-N2,N2-dimethylethanediamine (IQDMA), 5-(2-phenylpyrazolo [1,5-a]pyridine-3-yl)-1H-pyrazolo-[3,4-c]pyridazin-3-amine (FR180204), 1,4-diamino-2,3-dicyano-1,4-*bis*[2-aminophenylthio]butadiene (U0126), 2-(4-morpholin-yl)-8-phenyl-4H-1-benxopyrin-4-one (LY294002) and 1L6-hydroxymethyl-chrio-inosito-2-*O*-methyl-3-*O*-octadecyl-*sn*-glycero carbonate (HIMOC) were acquired from Calbiochem (San Diego, CA). The protein hormones used in our studies were dissolved in double-distilled water as 1 mM stocks and stored frozen at -80°C in small aliquots. The pharmacological inhibitors were prepared in a similar manner except that they were dissolved in DMSO as 10 mM stock solutions. During our experiments, frozen stocks of test substances were diluted with prewarmed culture medium to appropriate concentrations 15 min prior to drug treatment. The final dilutions of DMSO were always maintained at levels below 0.1% and did not affect the target gene expression in our cell culture system.

### Molecular cloning and tissue expression of carp activin βA and βB

To establish the structural identity of grass carp activin βA and βB, total RNA was isolated from the carp pituitary, reversely transcribed with SuperScript II (Invitrogen, Carlsbad, CA) and subjected to 3’/ 5’RACE using primers designed based on the conserved regions of respective gene targets in goldfish and zebrafish using a GeneRacer^™^ kit (Invitrogen). The full-length cDNAs for grass carp activin βA and βB were compiled using MacVector 9.5 (Oxford Molecular, Madison, WI). Sequence alignment, 3D protein modelling and phylogenetic analysis of carp activin βA and βB sequences obtained were conducted using CLUSTAL-W (http://www.ebi.ac.uk/Tools/msa/clustalw2), SWISS-MODEL (http://swissmodel.expasy.org/) and MEGA 6.0 (http://www.megasoftware.net), respectively. To examine the copy number of activin βA and βB gene in carp genome, Southern blot was also performed in genomic DNA isolated from grass carp blood cells after digestion with restriction enzymes including Pvu II, Sty I, Bgl II, EcoR V, Xba I, BamH I and Bgl I followed by hybridization with DIG-labelled cDNA probes for carp activin βA and βB as described previously [42].

For tissue expression profiling of the two activin β subunits, RT-PCR was carried out in total RNA prepared from selected tissues and brain areas with parallel PCR for β actin as the internal control. In this case, the RT samples prepared after DNase I digestion and reverse transcription of total RNA was subjected to PCR using primers for grass carp activin βA (5’ACATCCTCAACATGCTGCATCT3’ & 5’TCGTTGTAGTAGAGCATGGACA3’) and activin βB (5’ACCGACTGCAGATGAGAGAGAG3’ & 5’TCAAAGTAGAGCATGGACATGGTG3’) with PCR conducted for 35 cycles with denaturation at 94°C for 30 sec, annealing at 58°C for 45 sec and extension at 72°C for 30 sec. The authenticity of PCR products (947 bp and 911 bp for activin βA and βB, respectively) was routinely confirmed by PCR Southern using DIG-labelled probes for the respective gene targets. To characterize the size and form(s) of activin β transcripts expressed at the pituitary level, total RNA prepared from the carp pituitary was size-fractionated in 1% agarose gel and subjected to Northern blot using DIG-labelled riboprobes for carp activin βA and βB as described previously [37]. To test if the two activin β isoforms are expressed in a cell type-specific manner at the pituitary level, RT-PCR coupled with laser capture microdissection (LCM) of immuno-identified somatotrophs (GH cells), lactotrophs (PRL cells) and gonadotrophs (LH cells) was also performed. In cytospin preparation of carp pituitary cells, the respective cell types were identified by immunostaining with the antisera for carp GH (1:10,000), PRL (1:5,000) and LH (1:5,000) using Vectastain ABC kit (Vector Laboratories, Burlingame, CA) and isolated by LCM using a PixCell-II Cell Isolation System (Arcturus Engineering, Mountain View, CA) as reported previously [43]. The target cells captured (×25 or ×250 cells per LCM HS cap) were then subjected to DNase I digestion and reverse transcription followed by PCR using the same conditions with cycle number extended to 45 cycles with primers for activin βA and βB as described in preceding section. In this studies, RT-PCR with RT sample prepared from mixed populations of pituitary cells was used as the positive control and PCR for β actin was used as the internal control. To test for potential contamination of genomic DNA, PCR for target genes were also conducted in RNA samples without reverse transcription to serve as a negative control.

### Expression and functional testing of grass carp activin A and B

To confirm that the newly cloned cDNAs encode proteins with bioactivity, functional expression of grass carp activin A and B was performed in CHO cells. As a first step, the open reading frames (ORF) of carp activin βA and βB without 5’/3’ untranslated region (UTR) were PCR isolated and subcloned into pcDNA3.1(+) to generate the expression vector gc.Act βA for carp activin A and gc.Act βB for carp activin B, respectively. After that, the two vectors were transfected into CHO cells cultured in 35 mm dish with a seeding density of 0.5 × 10^6^ cells/dish separately using lipofectamine (Invitrogen). After a 6-hr transfection in OPTI-MEM medium, the old culture medium was replaced with DMEM medium supplemented with 10% FBS and the cell culture was maintained for 3 days under 5% CO_2_ and saturated humidity before the harvesting of the conditioned medium containing carp activin A or B released from CHO cells. To test the functionality of carp activin A and B, GH3 cells with endogenous expression of activin receptor [24] were maintained in Ham F-10 medium in 24-well plate with a seeding density of 0.1 × 10^6^ cells/well and transfected for 6 hr with lipofectamine in the presence of 0.2 μg/well of the firefly luciferase-expressing activin reporter pAR3-Lux [44], 0.05 μg/well FAST-2 expression vector (as a co-factor for activin induction) and 0.02 μg/well RL-TK.Luc (a renilla luciferase-expressing vector as internal control). After transfection, the cells were incubated for another 18 hr for recovery prior to drug treatment. For activin treatment, a 200 μ1 volume of the conditioned medium harvested from CHO cells transfected with gc.Act βA or gc.Act βB was added to individual wells of GH3 cells transfected with pAR3-Lux. Similar treatment with the conditioned medium from CHO cells transfected with the blank vector pcDNA3.1(+) was used as the control for the experiment. After 24-hr incubation with the conditioned media, GH3 cells were rinsed with 1 × PBS and lysed with passive lysis buffer (Promega, Madison, WI). Cell lysate prepared was then subjected to measurement of firefly and renilla luciferase activities with Dual-Glo Luciferase Assay (Promega) in a MicroLumat LB96V Luminometer (Berthold Tech, Bad Wildbad, Germany). In our studies with GH3 cells, co-treatment of the conditioned medium with follistatin, the activing-binding protein known to neutralize activin’s action, was also performed to confirm that the effects observed were indeed caused by actvin A/B released from CHO cells.

### GH regulation by activin and follistatin in grass carp pituitary cells

Primary culture of grass carp pituitary cells was prepared by trypsin/DNase II digestion method [35] and seeded at a density of 2.5 × 10^6^ cell/well in poly-D-lysine precoated 24-well plate. After overnight incubation to allow for recovery from enzyme digestion, static incubation with activin A and B was performed for 48 hr to examine their effects on GH regulation at the pituitary level. To investigate the functional role of actvin produced locally in pituitary cell culture, parallel treatment with follistatin to nullify the effects endogenous activin was also conducted. After drug treatment, culture medium was harvested to monitor the effect on GH release and the remaining cells were lysed in RIPA medium (50 mM Tris-HCl, 1% Nonidet-P40, 0.25% sodium deoxycholate, 1 mM EDTA and 150 mM NaCl) to prepare the cell lysate for detection of GH cell content. The protein contents of GH in culture medium and cell lysate were evaluated in a semi-quantitative manner by Western blot using antiserum raised against carp GH as described previously [36].

To correlate the results of GH cell content with its transcript expression, similar experiments were repeated to study the effects of activin and follistatin on GH mRNA expression. In this case, total RNA was isolated from pituitary cells after drug treatment, digested with DNase I to remove genomic DNA contamination, reversely transcribed and subjected to real-time PCR for GH mRNA measurement. To shed light on the mechanisms for GH mRNA regulation, the possible involvement of modification of transcript stability and transcriptional regulation of GH gene expression were also tested. To examine the transcript stability of GH mRNA, pituitary cells were treated with activin A and B in the presence of the transcriptional inhibitor actinomycin D (8 μM). The time course for the clearance of GH mRNA with/without activin treatment was used to deduce the half-life (*T*_*1/2*_) of GH transcript expressed in carp pituitary cells. To evaluate the functional role of GH gene transcription, the expression of GH primary transcript was monitored in pituitary cells with activin treatment using real-time PCR. For GH mRNA measurement, real-time PCR was conducted with RT samples at 1:100 dilution using primers flanking position 75 and 326 of grass carp GH cDNA (with intron I in between to monitor genomic DNA contamination). These primers could yield a PCR product of 252 bp in size with melting temperature (*Tm*) at 92.2°C for GH mature mRNA. In the case of GH primary transcript, undiluted RT samples were used for real-time PCR with primers covering position 348 and 557 of grass carp GH gene (covering the junction of exon II and intron II). These primers could selectively produce a PCR product of 210 bp in size with *Tm* of 91.4°C. The real-time PCR for GH mRNA and primary transcript were conducted with parallel measurement of 18S RNA (as the internal control) according to the procedures and PCR conditions as described previously [36].

### Activin modulation of GH promoter activity expressed in GH3 cells

To confirm that the results based on GH primary transcript could be correlated with corresponding changes in GH gene transcription, the 5’promoter (from position -986 to +13) of grass carp GH gene (GenBank No. X30419) was subcloned into the firefly luciferase-expressing reporter pGL3 (Promega) to generate the construct pGH(-986).LUC. The construct was used in transfection study with GH3 cells to examine the effects of activin A and B treatment on GH promoter activity. To delineate the promoter region with activin-responsive sequence, 5’deletion analysis of GH promoter with activin induction was conducted with pGH.LUC constructs carrying decreasing lengths of GH promoter from -986 to -446 generated by mung bean nuclease digestion, including pGH(-986).LUC, pGH(-761).LUC, pGH(-742). LUC, pGH(-656).LUC and pGH(-446).LUC. Given that the biological actions of activin are known to be mediated by SMAD pathway, especially via SMAD2 and SMAD3 [13], the effects of SMAD2 and SMAD3 over-expression on GH promoter activity were examined by co-transfection of GH3 cells with pGH(-986).LUC in the presence of the expression vector for mouse SMAD2/SMAD3 (cDNA Resource Center, University of Missouri). To test the possible involvement of SMAD2 and SMAD3 in activin regulation of GH promoter activity, activin A and B treatment were performed in GH3 cells following co-transfection of pGH(-986).LUC with the expression vector for the dominant negative (DN) mutant of SMAD2 or SMAD3 (Addgene, Cambridge, MA). The DN mutants of SMAD2 and SMAD3 were constructed with alanine substitution for all the serine residues in the SSXS domain and the mutated SMADs produced could serve as the competitive inhibitor for the respective SMADs to prevent their binding with activin receptors [45]. In these experiments, GH3 cells seeded at 0.1 × 10^6^ cells/well in 24-well plate were transfected for 6 hr using lipofectamine with 0.1 μg/well pGH(-986).LUC (or other pGH.LUC constructs with decreasing lengths of GH promoter), 0.02 μg/well RL-TK.Luc (the renilla luciferase-expressing vector as internal control) and 0.05 μg/well pcDNA3.1(+) (the blank vector for SMADs & their DN mutants, as carrier DNA for transfection). For the studies on the functional role of SMADs, appropriate amount of pcDNA3.1(+) used in transfection was replaced with the expression vectors for SMAD2/3 (7.5 to 30 ng/well) or their DN mutants (50 ng/well). After transfection, GH3 cells were treated with activin A and B for 24 hr prior to the preparation of cell lysate for luciferase activity measurement as described in the preceding section for functional testing of grass carp activin A and B.

### Activin and follistatin regulation by GH and LH in carp pituitary cells

To test if the intrapituitary activin/follistatin system can be regulated by pituitary hormones in the carp pituitary, carp pituitary cells (2.5 × 10^6^ cell/well in 24-well plate) were treated with GH, hCG, LH or FSH for the dose(s) and duration as indicated in individual experiments to study the effects on activin βA, activin βB and follistatin mRNA expression. To test the functional role of GH and LH produced locally in the cell culture, immunoneutralization to remove endogenous GH and LH was also performed using antisera raised against carp GH and LH, respectively. To study the possible interactions of GH and LH in regulating the activin/follistatin system at the pituitary level, GH modulation of activin βA, activin βB and follistatin gene expression were also monitored in carp pituitary cells with hCG co-treatment. In these studies, total RNA was isolated after drug treatment and the RT samples prepared were subjected to real-time PCR for activin βA, activin βB and follistatin mRNA using a LightCycler Master SYBR Green I Kit (Roche, Basel, Switzerland) with a RotorGene 6000 Real-time PCR System (Corbett, NSW, Australia). In these real-time PCR assays, gene-specific primers for carp activin βA (5’GCAGTAGTTAGCGTGATACCCAG3’ & 5’TGGACGGGGACAGCAGCTTTC3’, producing a 305 bp PCR product with *Tm* at 92°C), activin βB (5’ACCGACTGCAGATGAGAGAGA3’ & 5’ACAGGCTGGACTTAGATGGAGT3’, producing a 225 bp PCR product with *Tm* at 89°C) and follistatin (5’ATGCAAAGGGCACCCGGATCT3’ & 5’ATCGCATGACTTGGCCTTGATG3’, producing a 292 bp PCR product with *Tm* at 91°C) were used for quantitative PCR with serial dilutions of plasmid DNA carrying the ORF for the respective gene targets as the standards for data calibration. PCR reactions were conducted for 32 cycles with denaturation at 95°C for 30 sec, annealing at 56°C for 30 sec, and extension at 72°C for 30 sec. SYBR green signals for PCR products were recorded by the end of each cycle with acquiring temperature at 90°C, 86°C and 88°C for activin βA, activin βB and follistatin, respectively. Authenticity of PCR products were routinely confirmed by melting curve analysis after the real-time PCR assays. In our studies, parallel measurement of 18S RNA was also conducted to serve as the internal control.

### Data transformation and statistical analysis

For real-time PCR of target mRNA and primary transcript, standard curves constructed with the respective plasmid DNA standards with a dynamic range of ≥10^5^, amplification efficiency ≥ 98% and correlation coefficient ≥ 0.95 were used for data calibration under unsupervised mode with RotorGene Q software 1.7 (Corbett). Given that the levels of 18S RNA expressed in carp pituitary cells did not exhibit significant changes with drug treatment in our experiments, the raw data of real-time PCR (in femtomole target transcript per 10^6^ cells) were simply transformed as a percentage of the mean value of the target gene expression in the control group (as “%Ctrl”). To evaluate the clearance rate of GH transcript, the *T*_*1/2*_ value defined as the time required for GH mRNA reduced to 50% of its initial level was deduced using the one-phase exponential decay model with Prism 3.2 (GraphPad, San Diego, CA). For GH promoter studies, the data for firefly luciferase activity (in RLU unit) were transformed as a ratio of the renilla luciferase activity detected in the same sample (as “LUC activity ratio”), mainly to control for possible variation in transfection efficiency between wells. Data presented (Mean ± SEM) are pooled results from four separate experiments (N = 4) and were analysed either with Student’s t test or ANOVA followed by Newman-Keuls multiple comparison test. Differences between experimental groups were considered as significant at P < 0.05.

## Results

### Molecular cloning of grass carp activin βA and βB subunits

**Fig 1:**
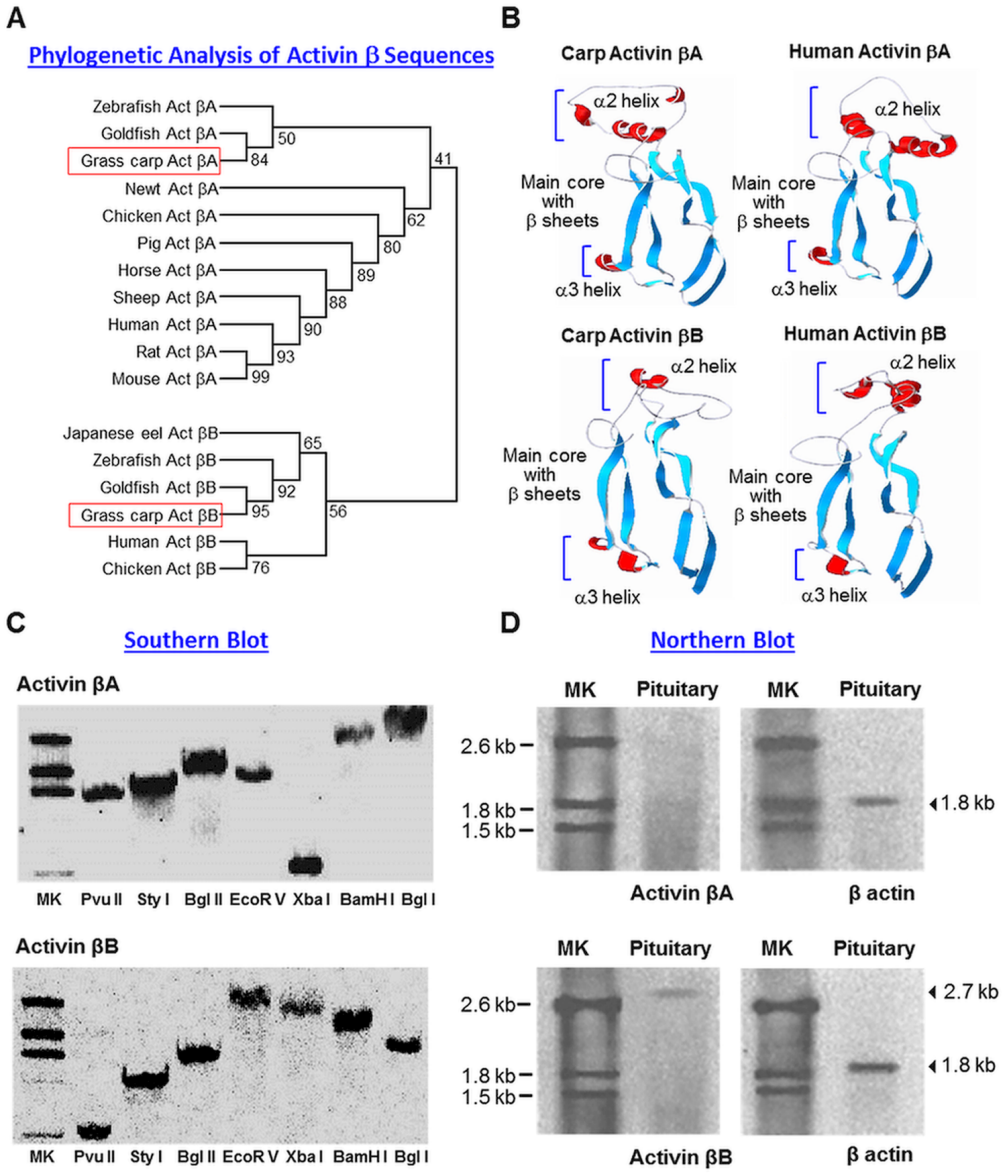
Sequence analysis, gene copy number and pituitary expression of grass carp activin βA and βB. (A) Phylogenetic analysis of carp activin βA and βB cDNA sequences with corresponding sequences reported in other species using neighbour-joining method with MEGA 6.0. The numbers presented with individual nodes of the guide tree are the percentage of bootstrap values based on a 1000 bootstraps. (B) 3D protein modelling of grass carp activin βA and βB using SWISS-MODEL and DeepView with the crystal structures of human activin βA and βB as the template, respectively. The regions in red represent the α2 and α3 helical domains while the two pairs of antiparallel β strands forming the main core of activin β subunit are marked in blue. (C) Gene copy number for activin βA and βB deduced by genomic Southern. Southern blot was conducted with DIG-labelled probes for activin βA and βB, respectively, in grass carp genomic DNA with prior digestion of the restriction enzymes as indicated. (D) Characterization of activin βA and βB transcripts expressed in the carp pituitary. Total RNA was isolated from the carp pituitary, resolved in 1% agarose gel and subjected to Northern blot with DIG-labelled probes for activin βA and βB. Parallel blotting for β actin mRNA was used as the internal control. (MK: Size markers for RNA transcripts).

Using 5’/3’RACE, the full-length cDNAs for grass carp activin βA (GenBank Accession No DQ 340763) and βB (GenBank Accession No DQ340764) were cloned. The activin βA cDNA was found to be 1559 bp in size composed of a 116 bp 5’UTR, 231 bp 3’UTR, and 1212 bp ORF encoding a 404 a.a. activin βA precursor with deduced MW of ~44 kDa (S1 Fig). In the case of activin βB, the cDNA was shown to be 2629 bp in size with a 273 bp 5’UTR, 1180 bp 3’UTR, and 1176 bp ORF encoding a 392 a.a. activin βB precursor with deduced MW of ~43 kDa (S2 Fig). To establish the evolutionary relationship of carp activin βA and βB with the corresponding nucleotide sequences reported in other species, phylogenetic analysis of these two newly cloned cDNAs based on neighbour-joining method was performed with MEGA 6.0. As shown in Fig 1A, grass carp activin βA and βB can be clustered into the clades of fish activin βA and βB, respectively, and are closely related to the corresponding sequences of other members of Cyprinid family including goldfish and zebrafish. Within the 3’UTR of both activin cDNAs, a canonical polyadenylation signal AATAAA could be noted, despite three additional atypical polyadenylation signals (TTTAAA or AAAAAG) were also found in the same region of activin βB. As revealed by SignalP 3.0 (http://cbs.dtu.dk/services/SignalP), a signal peptide with the size of 18 a.a. and 27 a.a. were also identified in the N-terminal of the deduced protein sequence of carp activin βA and βB, respectively. Presumably via the dibasic cleavage site (KR) located at the end of the pro-peptide region, the 161 a.a. mature peptides of activin βA and βB could be released by protein processing of the respective precursors. Of note, a N-linked glycosylation site (NVT or NIT) was also located in the pro-peptide of carp activin βA and βB, it raises the possibility that the precursors of the two activin β isoforms may be N-linked glycosylated.

Based on sequence alignment with CLUSTAL-W, the deduced a.a. sequences of grass carp activin βA (S3 Fig) and βB (S4 Fig) were found to be highly homologous to the corresponding sequences reported in other species, especially in the region of mature peptide (75%–99% and 93%–99% for activin βA & βB mature peptide from fish to mammals, respectively). Within the mature peptide, besides the TGFβ signature motif commonly found in TGFβ family members, the nine cysteine residues involved in intra/intermolecular disulphide bonding for structural stabilization and dimer formation were found to be well-conserved in the two activin β isoforms. Since the mature peptide sequences of carp activin βA and βB are highly comparable to their mammalian counterparts, 3D protein modelling with SWISS-MODEL and DeepView programs was conducted using the crystal structures of human activin βA and βB as the templates. As shown in Fig 1B, similar to the 3D protein models of human activin β subunits, activin βA and βB of carp origin were found to have the typical structure of two pairs of antiparallel β strands forming the main core of activin β covering the region with the nine cysteine residues. Of note, the N-terminal of carp and human activin β subunits did not form a helical structure constituting the α1 helical domain of TGFβ family [46], although the α2 and α3 helical domains could still be noted in the protein models of activin βA and βB. When compared to the human counterparts, minor modifications were also noted in α2 and α3 helical domains of carp activin β subunits, with subdivision of α2 helix of carp activin βA and α3 helix of carp activin βB into more helical segments and “contraction” of the helical segments in α2 helix of human activin βB into a single helix in the carp counterpart.

### Gene copy number and tissue expression of grass carp activin βA and βB

To shed light on the gene copy number of activin βA and βB in the carp genome, Southern blot was conducted in genomic DNA isolated from the whole blood of grass carp. As shown in Fig 1C, a single band was consistently detected in DNA samples predigested with Pvu II, Sty I, Bgl II, Ecor V, Xba I, BamH I and Bgl I after hybridization with DIG-labelled probes for carp activin βA and βB, respectively, implying that the two activin β isoforms are single-copy genes in carp model. For tissue expression of activin βA and βB, Northern blot was carried out in total RNA prepared from the carp pituitary (Fig 1D). In this case, a single band of ~2.7 kb in size could be noted after hybridization with the DIG-labelled probe for activin βB but not βA. The lack of hybridization signals for activin βA could not be due to RNA degradation during sample preparation as the corresponding signals for β actin (used as internal control) could still be observed in the same study.

**Fig 2:**
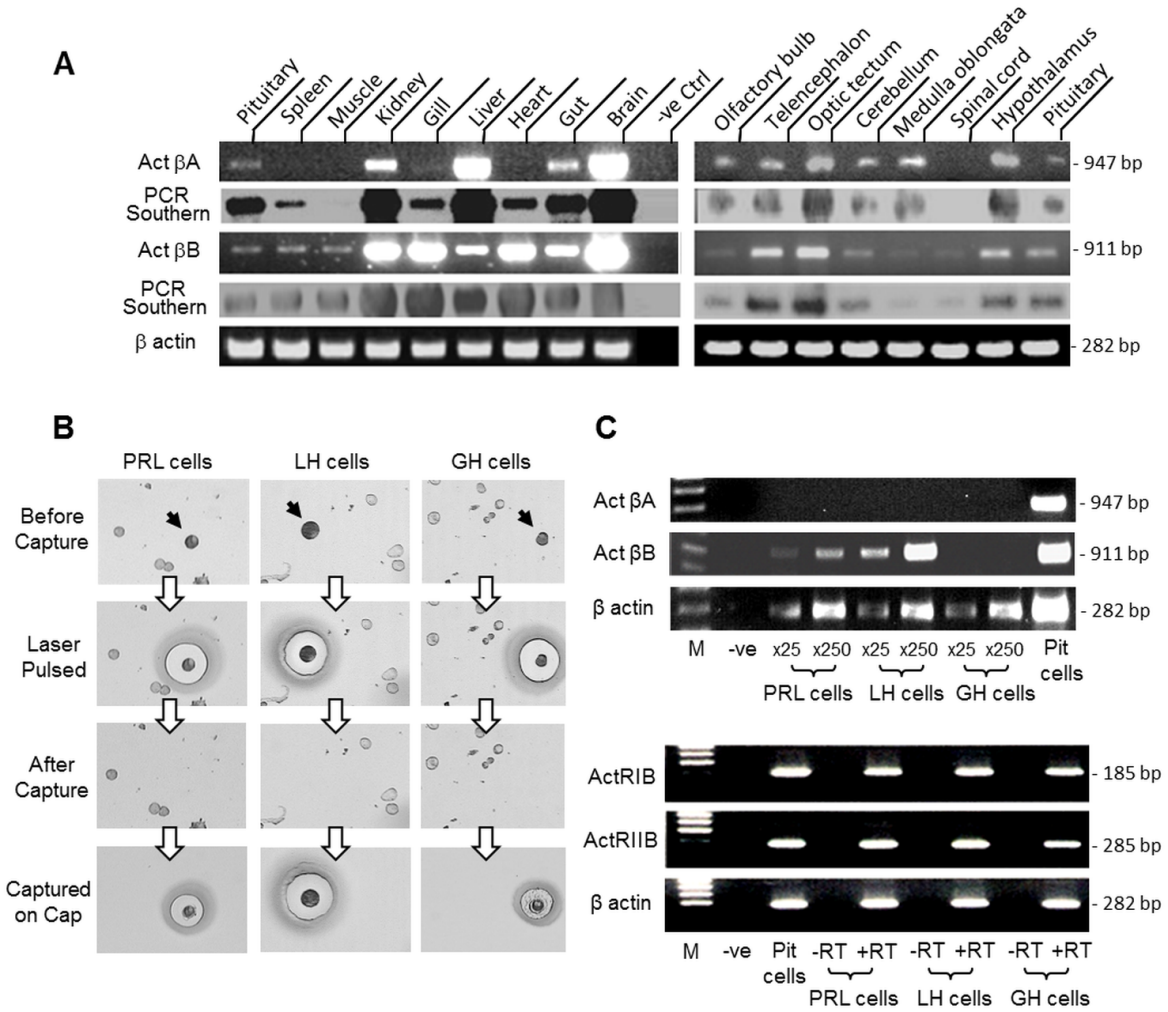
Tissue distribution of activin βA and βB expression in grass carp. (A) RT-PCR of activin βA and βB expression in selected tissues and brain areas. Total RNA was isolated from the tissues/brain areas as indicated and subjected to RT-PCR with primers specific for carp activin βA (Act βA) and βB (Act βB), respectively. The authenticity of PCR products was confirmed by PCR Southern with DIG-labelled probes for respective gene targets with parallel PCR for β actin as internal control. (B) LCM capture of immuno-identified lactotrophs (PRL cells), gonadotorphs (LH cells) and somatotrophs (GH cells) from grass carp pituitary cells. The three cell types (marked by black arrows) were identified by immunostaining using antisera for PRL, LH and GH, respectively. After pulsed with infrared laser, the target cells with signals for respective hormones were captured on LCM HS cap and subjected to RT-PCR with primers for different targets. (C) Expression of activin βA and βB as well as their receptors ActRIB and ActRIIB in immuno-identified pituitary cells. Pure populations of PRL, LH and GH cells captured on LCM HS cap (×25 or ×250 cells/cap) were used for RT-PCR with primers for carp activin βA and βB, respectively (upper panel). For detection of ActRIB and ActRIIB, RNA samples prepared from the respective cell types (×250 cells/cap) with reverse transcription (+RT) were used for RT-PCR using primers for carp ActRIB and ActRIIB, respectively (lower panel). Parallel PCR in RNA samples without reverse transcription (-RT) was used as negative control and PCR with the RT sample prepared from mixed populations of pituitary cells (Pit cells) was used as positive control. In these experiments, RT-PCR for β actin was routinely performed to serve as the internal control.

To further examine the tissue expression of activin βA and βB, a more sensitive approach, namely RT-PCR, was used (Fig 2A). Based on our validation, the PCR cycle number was fixed at 35 cycles for activin βA and βB detection, as it is in the mid-log phase of PCR amplification for the respective gene targets. In these studies, PCR signals for activin βB were found to be ubiquitously expressed in tissues including the pituitary, spleen, muscle, kidney, gills, liver, heart, gut and brain as well as in brain areas including the olfactory bulbs, telencephalon, optic tectum, hypothalamus, cerebellum, medulla oblongata and spinal cord. A similar distribution was also observed for the corresponding signals for activin βA, except that the βA signals were not detected in the muscle and spinal cord. Given that (i) pituitary cells can act as a regulatory target for activin [4] and (ii) cell type-specific expression of activin β has been reported in the pituitary of mammalian models [47, 48], the expression profiles of activin βA and βB as well as their receptors ActRIB and ActRIIB were also investigated by RT-PCR coupled to LCM isolation of carp lactotrophs (“PRL cells”), gonadotrophs (“LH cells”) and somatotrophs (“GH cells”) identified by immunostaining with respective antisera (Fig 2B). As revealed by Fig 2C, the PCR signal for activin βA was not detected in RT samples prepared from increasing numbers (×25 & ×250 cells) of pure populations of the three cell types isolated by LCM, although a notable signal for the same target could still be observed in the sample of mixed populations of pituitary cells (“Pit cells”, used as a positive control). In the same study, interestingly, the corresponding signals for activin βB could be noted in RT samples prepared from lactotrophs and gonadotrophs but not somatotrophs. Furthermore, the intensity for activin βB signals was found to be noticeably higher in gonadotrophs when compared to that of lactotrophs. In contrast to the cell-type specific expression of activin βA and βB, the PCR signals for activin receptors, including ActRIB and ActRIIB, were readily detectable in RT samples for lactotorphs, gonadotriphs and somatotrophs. Since the PCR signals for activin receptors were absence in the samples prepared without RT reaction (“-RT”, used as negative control), the possibility that our target gene signals were the results of genomic DNA contamination is highly unlikely.

### Expression and functional testing of grass carp activin A and B

**Fig 3:**
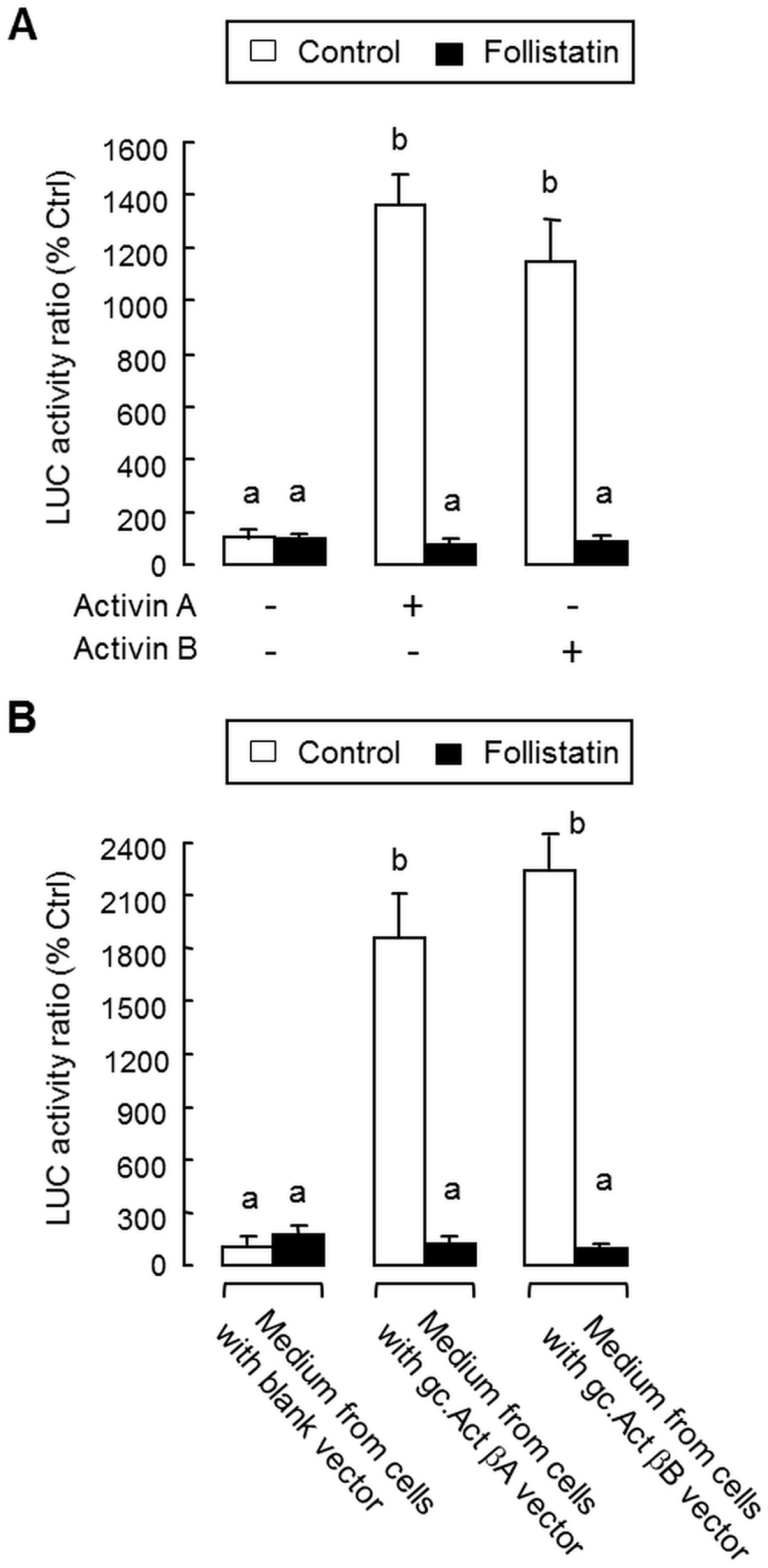
Functional testing of grass carp activin A and B in GH3 cells. (A) Validation of actvin responsiveness in GH3 cells with pAR3-Lux transfection. GH3 cells were transfected with the activin-responsive pAR3-Lux reporter and challenged for 24 hr with human activin A (10 ng/ml) and B (10 ng/ml), respectively. To confirm that the effects observed were specific for activin, parallel experiments were conducted with activin A and B induction in the presence of human follistatin (300 ng/ml). (B) Effects of carp activin A and B on pAR3-Lux reporter activity expressed in GH3 cells. Conditioned media obtained from CHO cells transfected with the expression vectors for carp activin βA (gc.Act βA) and βB (gc.Act βAB) were used as the source of carp activin A and B, respectively. For functional testing of carp activins, GH3 cells with pAR3-Lux transfection were treated for 24 hr with the conditioned media containing activin A and B, respectively, with parallel treatment of conditioned medium harvested from CHO cells transfected with the blank vector pcDNA3.1(+) as the control. To confirm that the effects on pAR3-Lux reporter activity were indeed mediated by activin, parallel studies with the respective conditioned media were also repeated with co-treatment of follistatin (300 ng/ml). In these experiments, cell lysate was prepared from GH3 cells after drug treatment and used for luciferase (LUC) activity measurement using a Dual-Glo luciferase assay. Data presented are expressed as Mean ± SEM (N = 4) and the groups denoted by different letters represent a significant difference at P < 0.05 (ANOVA followed by Newman-Keuls test).

To confirm that the newly cloned cDNAs for activin βA and βB indeed encode functional proteins of the respective gene targets, functional expression was conducted in CHO cells with transfection of the carp activin βA and βB expression vectors gc.Act βA and gc.Act βB, respectively. After cultured for 3 days, the conditioned media with carp activin A and B released from CHO cells were harvested and their bioactivity was tested in GH3 cells transfected with the luciferase-expressing activin reporter pAR3-Lux carrying tandem repeats of activin-responsive elements in its 5’ promoter. GH3 cells were selected for our studies as it is a somatotroph cell line with activin receptor expression and known to be highly responsive to activin induction [24]. In our validation, treatment with human activin A (10 ng/ml) and B (10 ng/ml) were both effective in elevating luciferase activity expressed in GH3 cells and these stimulatory effects could be blocked by co-treatment with human follistatin (300 ng/ml), the activin-binding protein known to neutralize activin’s action (Fig 3A), indicating that our assay system is fully functional for probing the bioactivity mediated by activins. In parallel experiments with GH3 cells transfected with pAR3-Lux, treatment with the conditioned media obtained from CHO cells transfected with gc.Act βA and gc.Act βB, respectively, was capable of triggering notable rises in luciferase activity expression (Fig 3B). However, similar treatment with conditioned medium from CHO cells transfected with the blank vector was without effects. Similar to the results for human activin A and B treatment, the stimulatory actions of conditioned media prepared by transfection with gc.Act βA and gc.Act βB were found to be totally abolished by co-treatment with follistatin (300 ng/ml). These results, taken together, suggest that the carp activin A and B released from CHO cells could induce transactivation of the activin-responsive promoter in pAR3-Lux and their stimulatory effects could be negated by the activin-binding protein follistatin.

### GH regulation by activin A and B in grass carp pituitary cells

**Fig 4:**
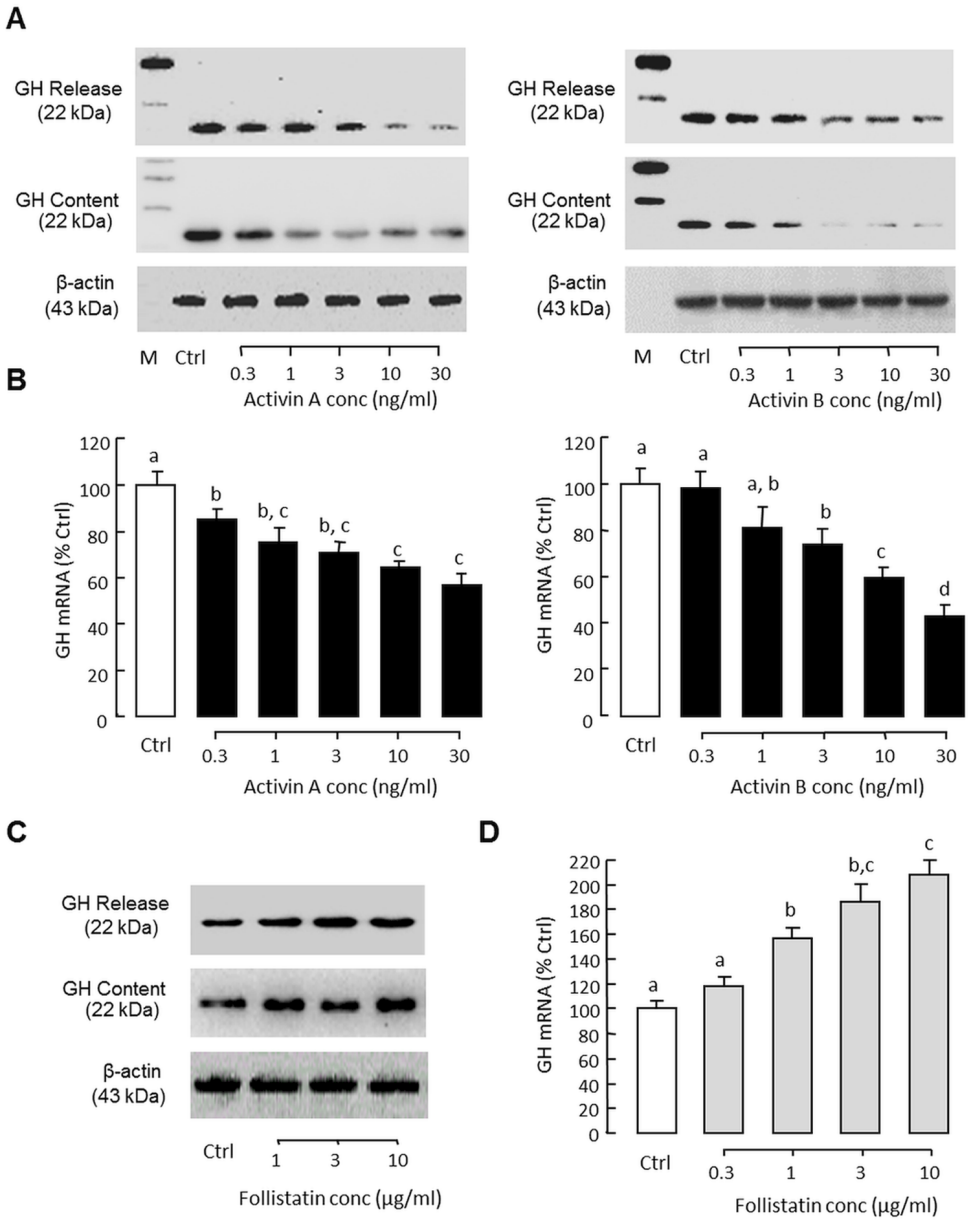
GH regulation by activin and follistatin in grass carp pituitary cells. Effects of increasing doses of human activin A and B on (A) GH secretion and GH cell content, and (B) GH mRNA expression in carp pituitary cells. In parallel experiments, the effects of removing endogenous activin with increasing levels of human follistation on (C) GH secretion and GH cell content, and (D) GH mRNA expression were also examined. In these studies, the duration of drug treatment was fixed at 48 hr. After treatment, culture medium (for GH release) and cell lysate (for GH cell content) were harvested and subjected to Western blot with GH antiserum with parallel blotting of β actin expression as the internal control. In separate experiments, total RNA was also isolated for real-time PCR of GH mature mRNA. GH mRNA data presented (Mean ± SEM) are pooled results from four separate experiments (N = 4) and the groups denoted by different letters represent a significant different at P < 0.05 (ANOVA followed by Newman-Keuls test).

**Fig 5:**
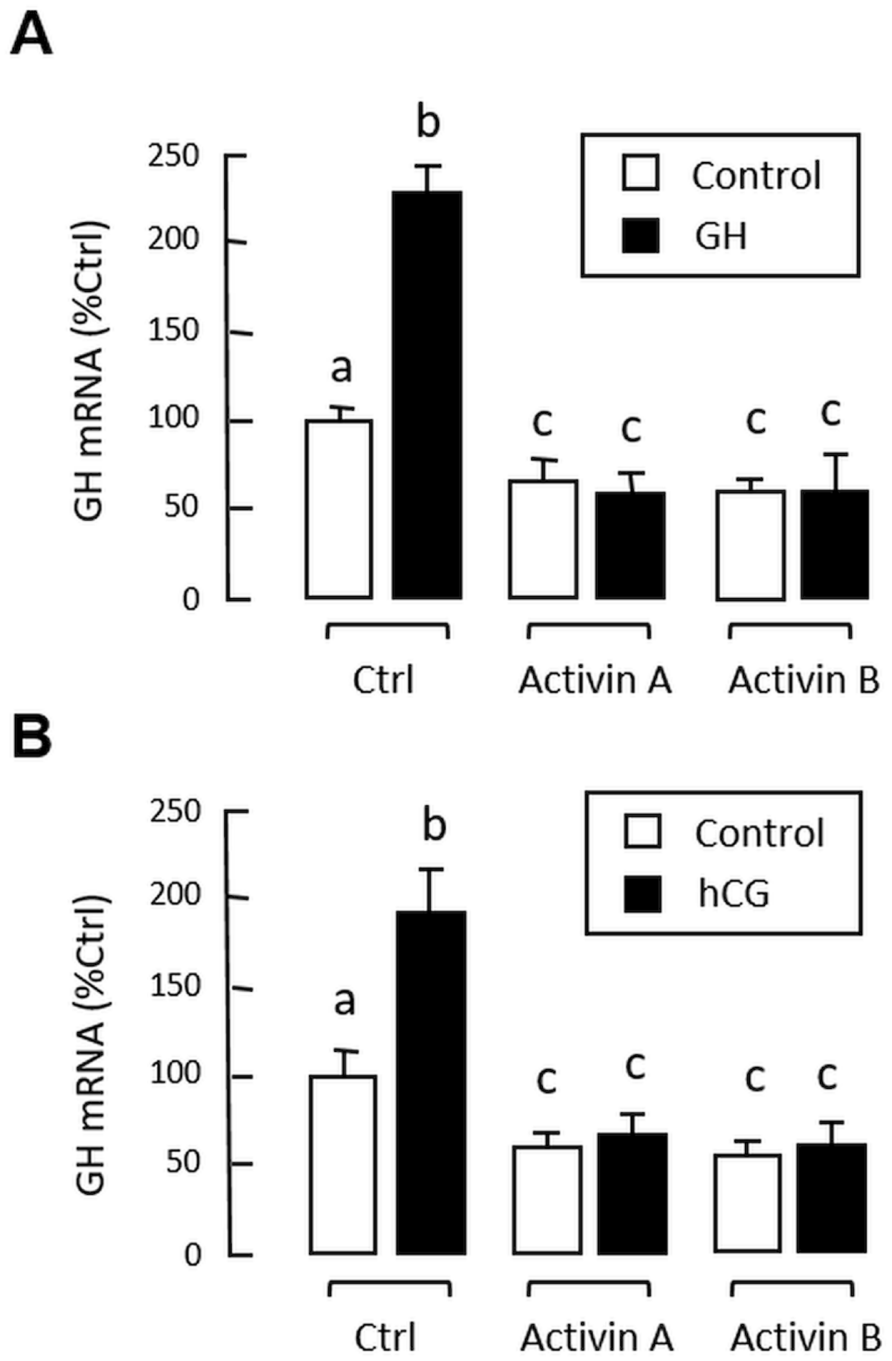
Inhibitory actions of activin on GH- and hCG-induced GH gene expression in carp pituitary cells. (A) Activin A and B treatment on GH-induced GH mRNA expression. Pituitary cells were treated for 48 hr with porcine GH (100 ng/ml) in the presence of human activin A (30 ng/ml) and B (30 ng/ml), respectively. (B) Activin A and B treatment on hCG-induced GH mRNA expression. Pituitary cells were exposed to hCG (40 IU/ml) for 48 hr with/without the co-treatment of human activin A (30 ng/ml) and B (30 ng/ml), respectively. After drug treatment, total RNA was isolated and subjected to real-time PCR for GH mRNA measurement. Data presented are pooled results from four separate experiments (N = 4) and the groups denoted by different letters represent a significant different at P < 0.05 (ANOVA followed by Newman-Keuls test).

To shed light on the functional role of activin/follistatin system in GH regulation in the carp pituitary, the effects of activin A and B on GH synthesis and secretion were examined in primary culture of grass carp pituitary cells. In this case, treatment with increasing levels (0.3–30 ng/ml) of human activin A and B for 48 hr were found to suppress GH release and GH cell content in carp pituitary cells (Fig 4A) with parallel drop in GH mRNA expression in a concentration-dependent manner (Fig 4B). However, the opposite effects were observed by “soaking up” endogenous activin with increasing levels of human follistatin (1–30 ng/ml, Fig 4C and 4D). In the carp pituitary, local release of GH (by autocrine actions) [36] and LH (by paracrine actions) is known to stimulate GH gene expression [40]. To study the possible interactions of activin with GH regulation by GH and LH, respectively, the effects of porcine GH and hCG (a functional analogue of LH) on GH mRNA expression were tested in carp pituitary cells with activin co-treatment. As shown in Fig 5A and 5B, activin A (30 ng/ml) and B (30 ng/ml) treatment not only could suppress basal but also block GH mRNA expression induced by GH (100 ng/ml) and hCG treatment (40 IU/ml).

**Fig 6:**
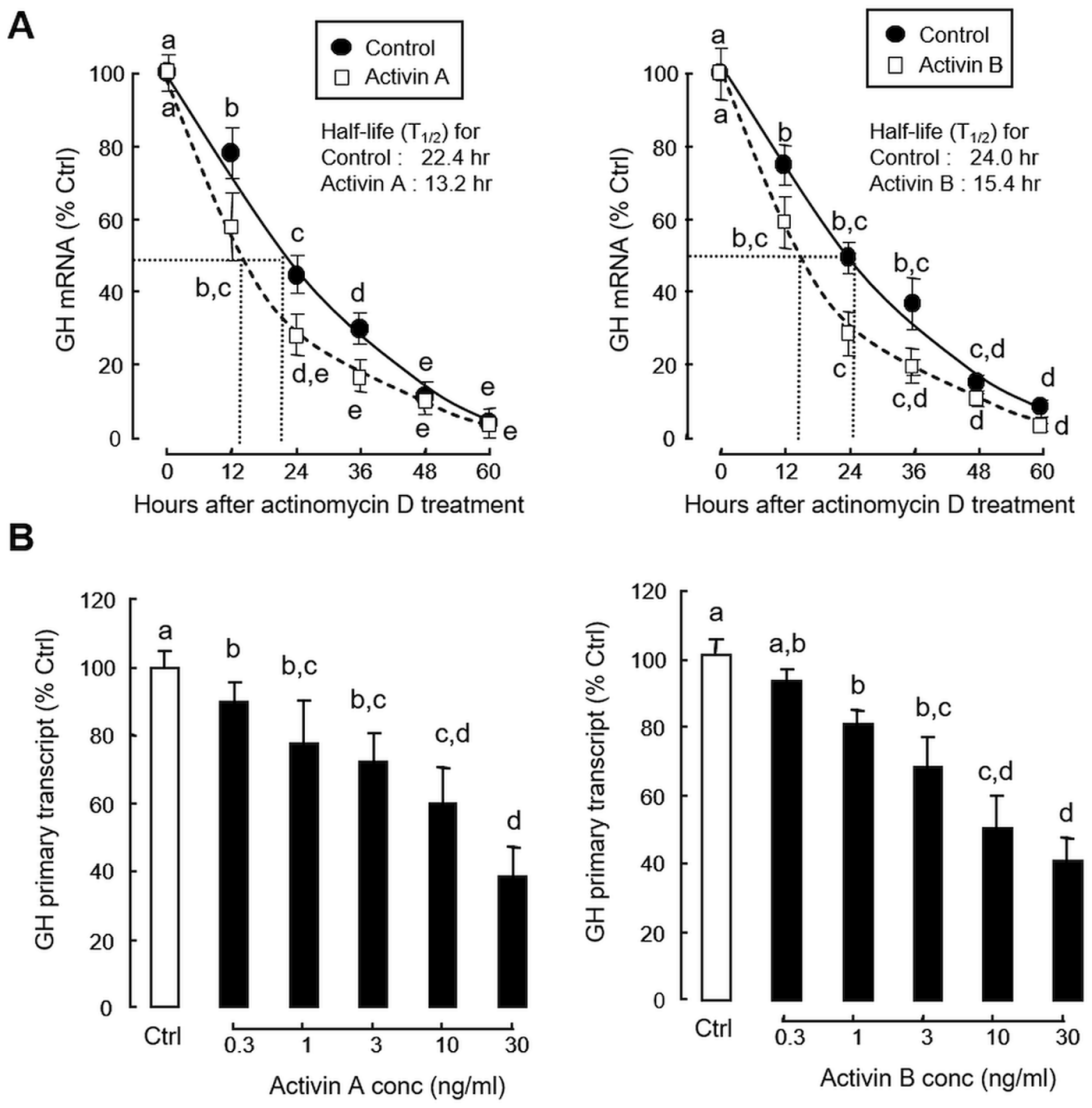
Inhibitory effects of activin on GH transcript stability and GH primary transcript expression. (A) Activin A and B treatment on GH mRNA clearance at pituitary level. Pituitary cells were treated with the transcriptional inhibitor actinomycin D (8 μM) in the presence or absence of activin A (30 ng/ml) or B (30 mg/ml). Total RNA was isolated at the respective time points as indicated and used for GH mRNA measurement by real-time PCR. The half-life (*T*_1/2_) of GH transcript, defined as the time required for target mRNA to drop to 50% of its initial value, was deduced from the respective GH mRNA clearance curves constructed based on the data obtained. (B) Activin A and B on GH primary transcript expression in carp pituitary cells. Pituitary cells were treated for 48 hr with increasing doses of activin A and B, respectively. After that, total RNA was isolated, digested with DNase I, and used in real-time PCR specifically developed for measurement of GH primary transcript. Individual groups denoted by different letters represent a significant different at P < 0.05 ((ANOVA followed by Newman-Keuls test).

To further evaluate the mechanisms involved in the drop of GH mRNA levels induced by activin, the transcript stability of GH mRNA expressed in carp pituitary cells was monitored in the presence of the transcriptional inhibitor actinomycin D (8 μM). By blocking gene transcription in cell culture, the clearance rate of GH mRNA was found to be enhanced by activin A (30 ng/ml) and B (30 ng/ml) treatment as reflected by the drop in *T*_*1/2*_ values for GH transcript revealed by our time-course studies (22.4 & 24.0 hr for the control *versus* 13.2 hr for activin A & 15.4 hr for activin B treatment, Fig 6A). Since the level of primary transcript in general can be taken as an index for target gene transcription [49], the possible role of GH gene transcription in the pituitary actions of activin was also examined by monitoring GH primary transcript expression (Fig 6B). Similar to the studies on GH mRNA expression, increasing levels (0.3–30 ng/ ml) of activin A and B were found to induce a dose-dependent inhibition on basal expression of GH primary transcript in carp pituitary cells.

### Activin A and B on GH promoter activity expressed in GH3 cells

**Fig 7:**
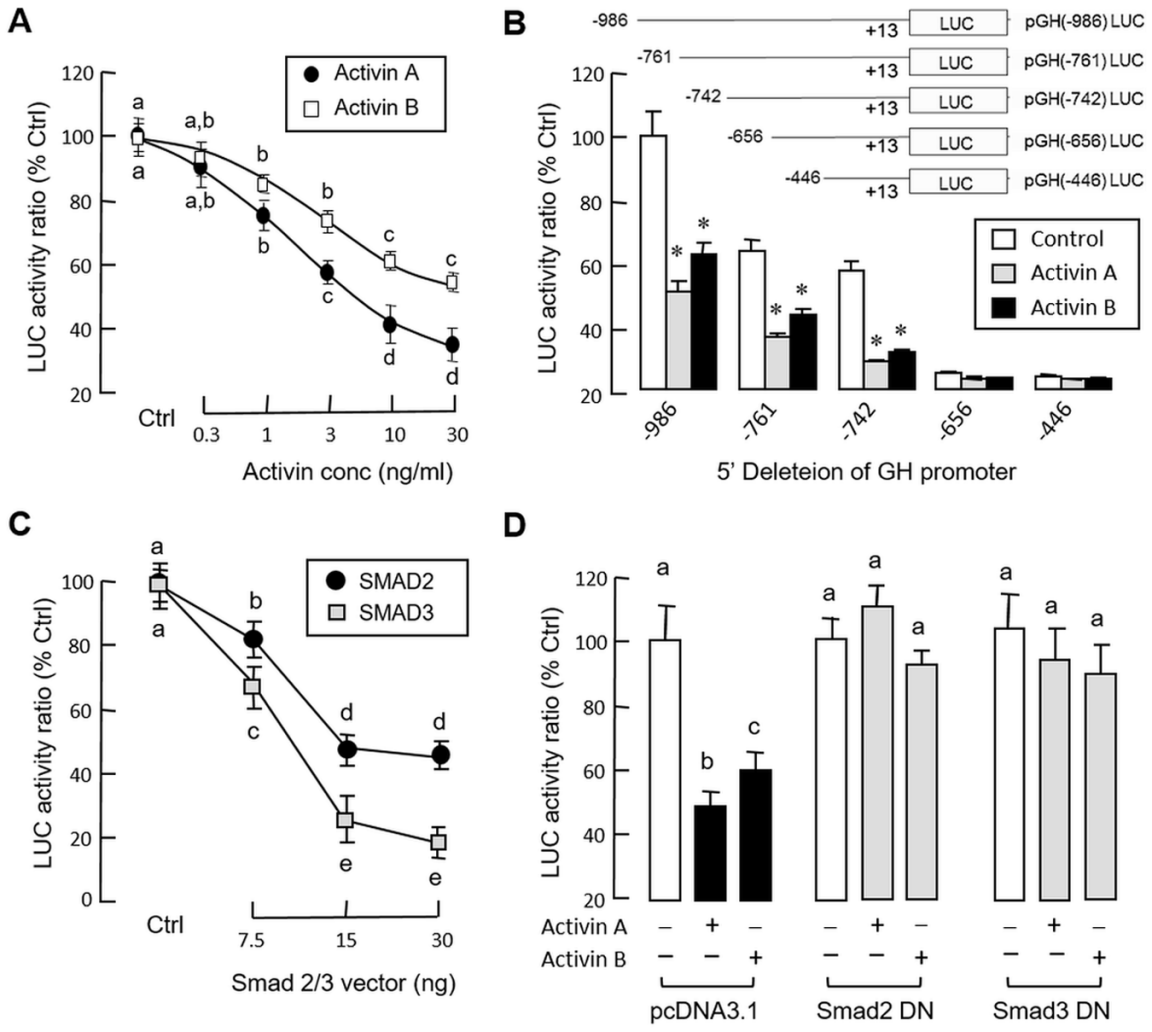
Inhibitory effects of activin and SMAD2/3 on GH promoter activity expressed in GH3 cells. (A) Activin A and B treatment on GH promoter activity expressed in GH3 cells. After pGH(-986).LUC transfection, GH3 cells were challenged for 24 hr with increasing doses of activin A and B as indicated. (B) 5’ Deletion analysis of GH promoter to delineate the activin-responsive region. GH3 cells were transfected with LUC reporters carrying decreasing lengths of GH promoter as indicated and treated for 24 hr with activin A (30 ng/ml) and B (30 ng/ml), respectively. (C) SMAD2 and SMAD3 expression on GH promoter activity expressed in GH3 cells. GH3 cells were transfected with pGH(-986).LUC in the presence of increasing doses of the expression vectors for SMAD2 and SMAD3, respectively. (D) Expression of dominant negative (DN) mutants of SMAD2 and SMAD3 on activin inhibition of GH promoter activity expressed in GH3 cells. GH3 cells were transfected with pGH(-986).LUC in the presence of the expression vector for SMAD2 DN mutant (Smad2 DN) or SMAD3 DN mutant (Smad3 DN). Parallel transfection with pcDNA3.1, the blank vector for DN mutants, was used as the control treatment. After transfection, the cells were challenged for 24 hr with activin A (30 ng/ml) and B (30 ng/ml), respectively. In these studies, cell lysate was prepared after drug treatment/SMAD expression and used for measurement of luciferase (LUC) activity with a Dual-Glo luciferase assay. Data presented for LUC activity are pooled results from four separate experiments and individual groups denoted by different letters (ANOVA followed by Newman-Keuls test) or marked with asterisks (Student’s t test comparing to the respective control) represent a significant difference at P < 0.05.

To elucidate the mechanisms for activin regulation of GH gene transcription at the promoter level, transfection studies were performed in GH3 cells with pGH(-986).LUC carrying the 986 bp 5’ promoter of grass carp GH gene. As shown in Fig 7A, treatment with increasing levels (0.3–30 ng/ml) of activin A and B were both effective in reducing luciferase activity expression in a dose-related fashion. 5’ Deletion analysis of GH promoter from position -986 to -446 also revealed that the inhibitory actions of activin A (30 ng/ml) and B (30 ng/ml) on luciferase activity expression could still be observed with promoter truncation from position -986 to -742 (Fig 7B). However, further deletion of GH promoter from position -742 to -656 and all the way to -446 could lead to a total loss of activin inhibition, despite a notable drop in basal luciferase activity was also observed. Of note, as reflected by promoter activation by forskolin or PACAP induction, the minimal promoter of grass carp GH gene could be mapped to the region downstream of position -88 (Data not shown). Therefore, the loss of inhibitory actions by activin A and B treatment could not be due to “excessive truncation” leading to the deletion of the minimal promoter. In mammals, the biological effects of activin are mediated by SMAD pathway coupled to activin receptors, especially via SMAD2 and SMAD3 [13]. In our study, co-transfection of GH3 cells with pGH(-986).LUC in the presence of increasing levels (7.5–30 ng/well) of the expression vectors for SMAD2 and SMAD3, respectively, were found to inhibit luciferase activity expression in a dose-dependent manner (Fig 7C). In parallel experiments, the inhibitory effects of activin A (10 ng/ml) and B (10 ng/ml) on luciferase activity expressed in GH3 cells could be reverted by co-transfection with the expression vectors for the DN mutants of SMAD2 (50 ng/well) and SMAD3 (50 ng/well), respectively (Fig 7D).

### Activin and follistatin regulation by GH in carp pituitary cells

**Fig 8:**
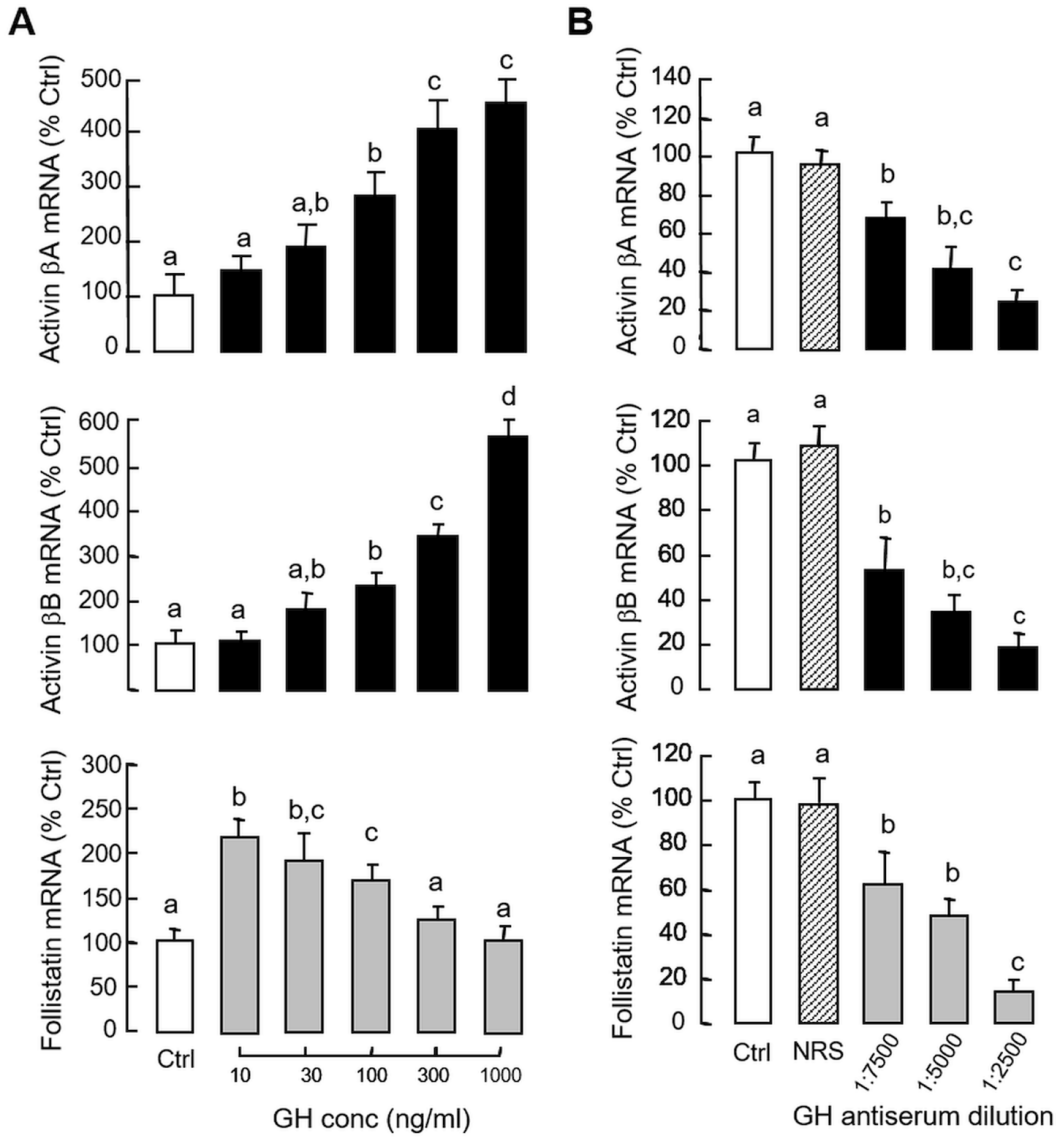
Regulation of activin and follistatin expression by local release of GH in carp pituitary cells. (A) GH treatment on activin βA, activin βB and follistatin mRNA expression at pituitary level. Pituitary cells were challenged for 24 hr with increasing concentrations of porcine GH as indicated. (B) Removal of endogenous GH by immunoneutralization on activin βA, activin βB and follistatin mRNA expression in carp pituitary cells. Pituitary cells were incubated for 24 hr with increasing levels of antiserum raised against carp GH and parallel treatment with normal rabbit serum (NRS, 1:2500) was used as a negative control. In these experiments, total RNA was isolated after drug treatment and used for real-time PCR measurement of activin βA, activin βB and follistatin mRNA, respectively. Data presented are pooled results from four separate experiments and individual groups denoted by different letters represent a significant different at P < 0.05 (ANOVA followed by Newman-Keuls test).

**Fig 9:**
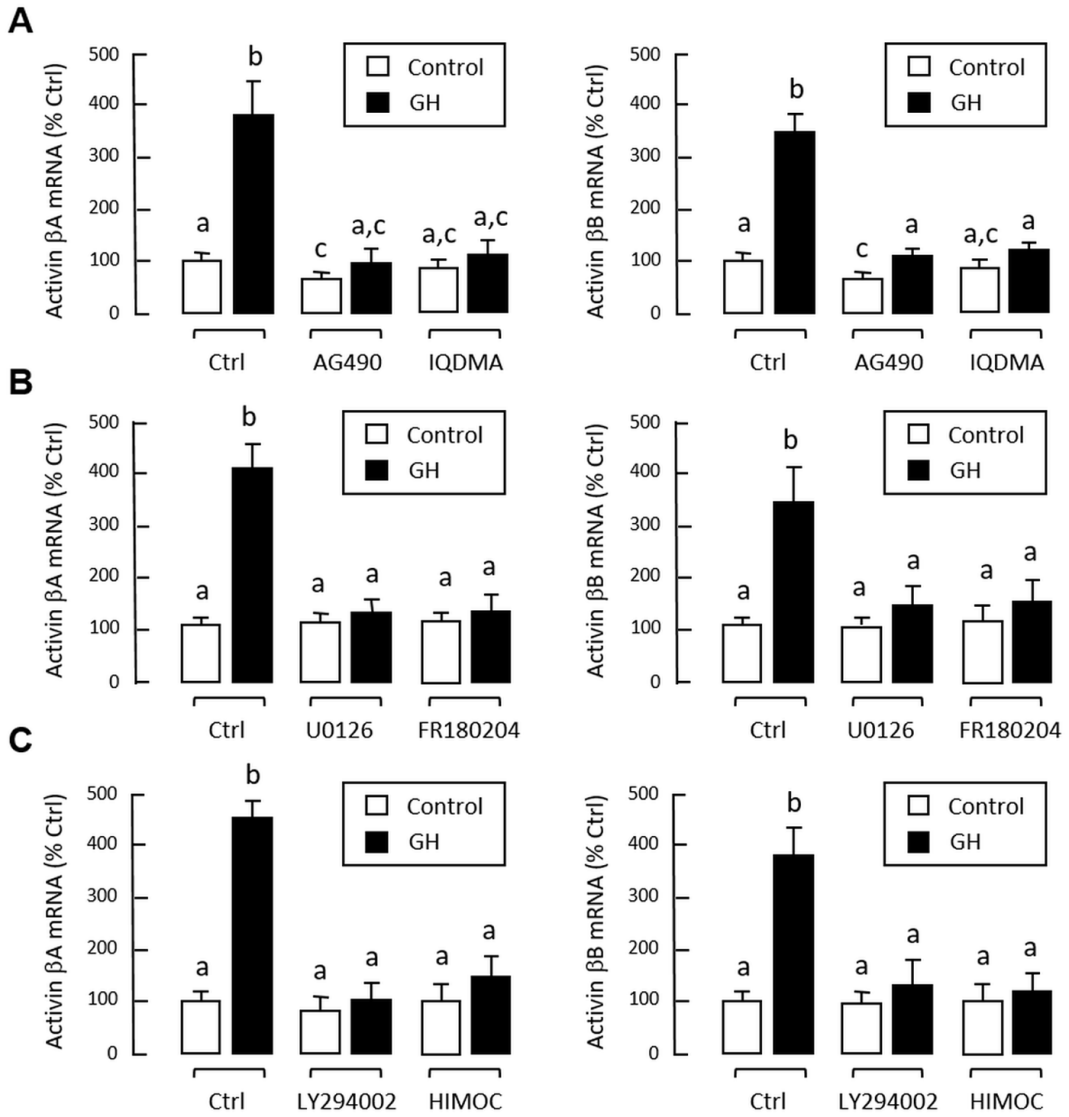
Signal transduction for GH-induced activin βA and βB gene expression in carp pituitary cells. Pituitary cells were treated for 24 hr with porcine GH (300 ng/ml) in the presence or absence of (A) the JAK_2_ inhibitor AG490 (400 μM) or STAT_5_ blocker IQDMA (50 μM), (B) MEK_1/2_ inhibitor U0126 (10 μM) or ERK_1/2_ inactivator FR180204 (2 μM), and (C) PI3K inhibitor LY294002 (10 μM) or Akt blocker HIMOC (20 μM). After treatment, total RNA was isolated and subjected to real-time PCR of activin βA (right panels) and βB mRNA (left panels), respectively. Data presented are pooled results from four experiments and individual groups denoted by different letters represent a significant different at P < 0.05 (ANOVA followed by Newman-Keuls test).

In preceding studies, activin A and B were shown to inhibit GH gene expression via direct action acting at the pituitary level. To test for reciprocal regulation of activin/follistatin system by GH release in carp pituitary, grass carp pituitary cells were challenged for 24 hr with increasing concentrations of GH (10–1000 ng/ml). In this case, GH treatment was found to up-regulate activin βA and βB mRNA levels in a dose-related fashion (Fig 8A). In parallel experiment, transcript expression of activin βA and βB were reduced dose-dependently by removing endogenous GH with immunoneutralization using increasing levels of antiserum raised against carp GH (Fig 8B). Although GH treatment could also elevate follistatin mRNA expression in carp pituitary cells, the stimulatory effect was observed only with lower doses of GH induction (10–100 ng/ml, Fig 8A). Apparently, the cells became refractory to GH at high doses (300–1000 ng/ml), suggesting that intrinsic mechanisms for down-regulation of follistatin gene expression may exist at the pituitary level. Similar to the corresponding responses for activn βA and βB, follistatin mRNA level could be down-regulated in a dose-dependent manner by immunoneutralization with increasing levels of GH antiserum (Fig 8B). In mammals, GH receptor is known to be functionally coupled with JAK_2_/STATs, MAPK and PI3K/Akt pathways [50]. To examine the post-receptor signalling for GH-induced activin βA and βB mRNA expression, a pharmacological approach was used with inhibitors for individual components of JAK_2_/STAT_5_ (Fig 9A), MEK_1/2_/ERK_1/2_ (Fig 9B) and PI3K/Akt pathways (Fig 9C). In this case, the stimulatory effects on activin βA and βB mRNA expression in carp pituitary cells induced by GH induction (300 ng/ml) were found to be notably suppressed/negated by co-treatment with the JAK_2_ inhibitor AG490 (100 μM), STAT_5_ blocker IQDMA (50 μM), MEK_1/2_ inhibitor U0126 (10 μM), ERK_1/2_ inactivator FR180204 (2 μM), PI3K blocker LY294002 (10 μM) and Akt inhibitor HIMOC (20 μM), respectively.

### Activin and follistatin regulation by hCG in carp pituitary cells

**Fig 10:**
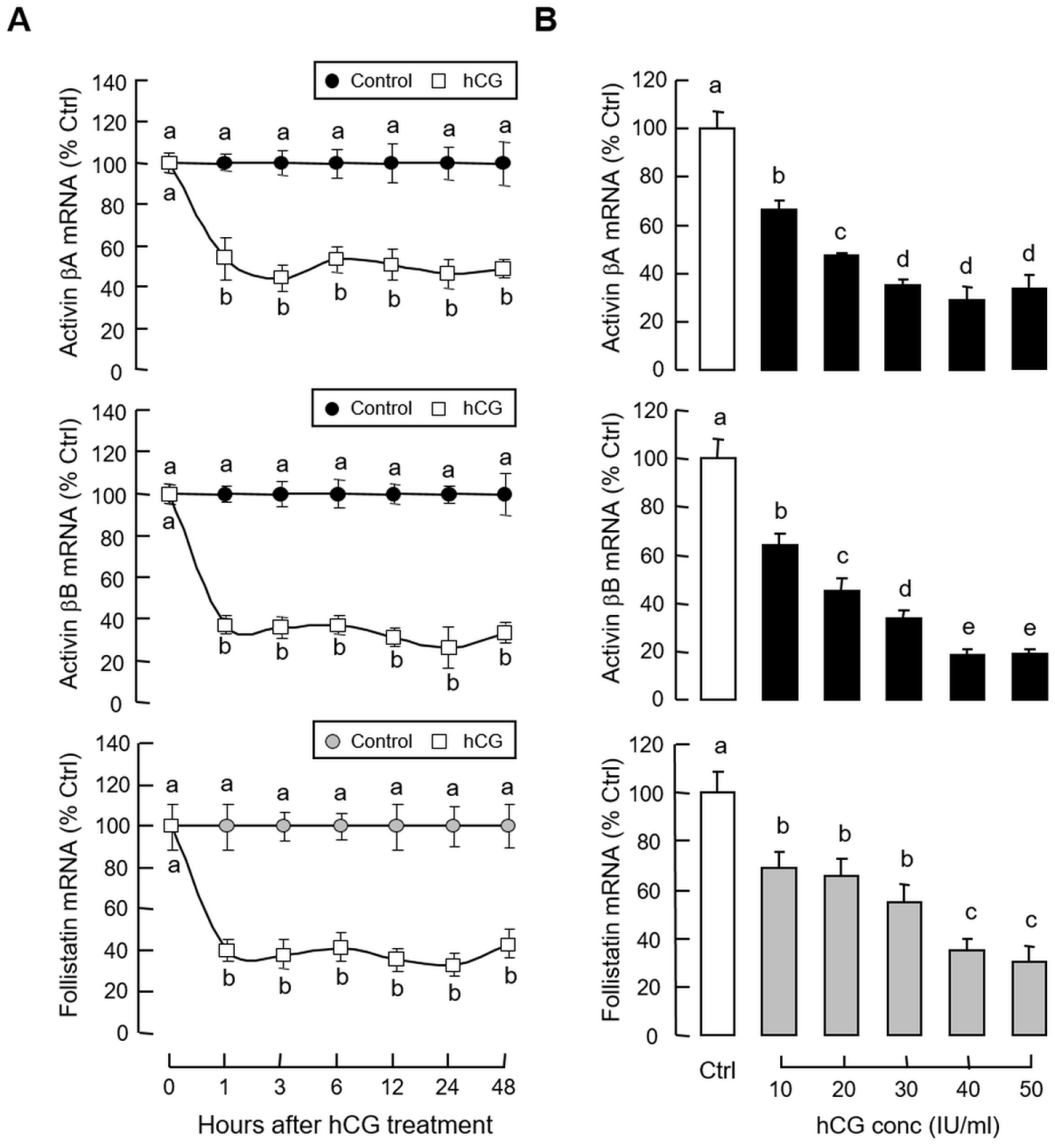
Effects of hCG on activin and follistatin expression in carp pituitary cells. (A) Time course and (B) dose dependence of hCG treatment on activin βA, activin βB and follistatin mRNA expression at pituitary level. Pituitary cells were treated with hCG (40 IU/ml) for the duration as indicated for the time course experiment. To test for dose dependence, the duration of drug treatment was fixed at 24 hr with cells exposed to increasing levels of hCG. After drug treatment, total RNA was isolated and used for real-time PCR measurement of activin βA, activin βB and follistatin mRNA, respectively. Data presented are pooled results from four experiments and individual groups denoted by different letters represent a significant different at P < 0.05 (ANOVA followed by Newman-Keuls test).

**Fig 11:**
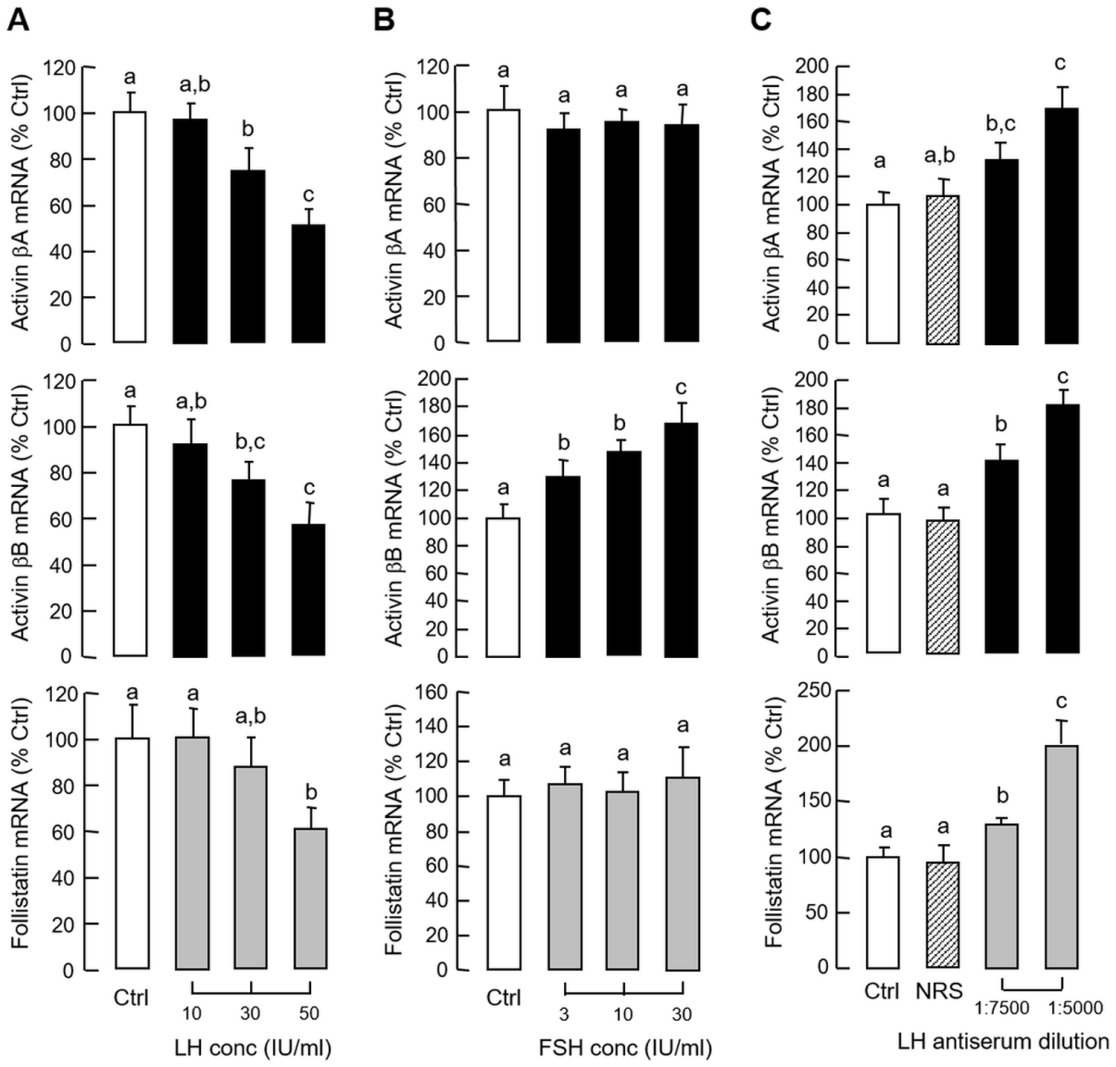
Regulation of activin and follistatin expression by local release of LH in carp pituitary cells. (A) LH and (B) FSH treatment on activin βA, activin βB and follistatin mRNA expression at pituitary level. Pituitary cells were treated for 24 hr with increasing levels of equine LH and FSH as indicated. (C) Removal of endogenous LH by immunoneutralization on activin βA, activin βB and follistatin mRNA expression in carp pituitary cells. Pituitary cells were incubated for 24 hr with increasing levels of antiserum raised against carp LH and parallel treatment with NRS (1:5000) was used as a negative control. In these experiments, total RNA was isolated after drug treatment and used for real-time PCR measurement of activin βA, activin βB and follistatin mRNA, respectively. Data presented are pooled results from four experiments and individual groups denoted by different letters represent a significant different at P < 0.05 (ANOVA followed by Newman-Keuls test).

**Fig 12:**
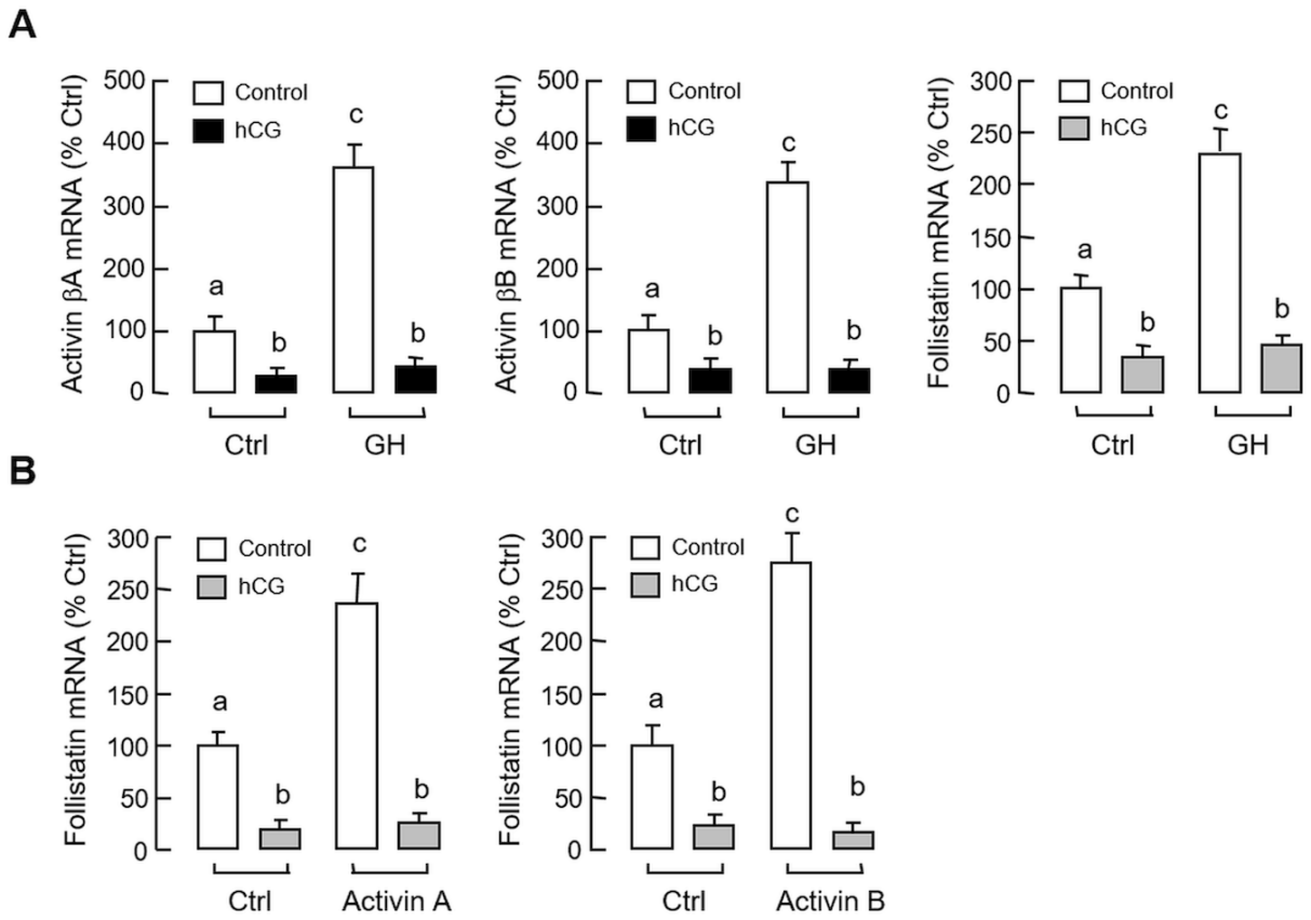
Inhibition of GH-induced activin and follistatin expression and activin feedback via follistatin by hCG treatment. (A) Effects of hCG on GH-induced activin βA, activin βB and follistatin mRNA expression at pituitary level. Pituitary cells were treated for 24 hr with GH (300 ng/ml) in the presence or absence of hCG co-treatment (40 IU/ml). (B) Effects of hCG on follistatin mRNA expression induced by activin A and B in carp pituitary cells. Pituitary cells were exposed to activin A (30 ng/ml) or B (30 ng/ml) for 24 hr with or without hCG co-treatment (40 IU/ml). In these experiments, total RNA was isolated after treatment and subjected to real-time PCR for the respective gene targets. Data presented are pooled results from four experiments and individual groups denoted by different letters represent a significant different at P < 0.05 (ANOVA followed by Newman-Keuls test).

Given that (i) LH and GH can interact in an autocrine/paracrine manner to up-regulate GH gene expression in the carp pituitary [41], and (ii) GH mRNA responses induced by GH and hCG in carp pituitary cells could be blocked by activin A and B (Fig 5), the functional role of LH in regulating activin and follistatin expression at the pituitary level was also examined. In grass carp pituitary cells, treatment with hCG (40 IU/ml) for various duration (up to 48 hr, Fig 10A) or with increasing concentrations (10–50 IU/ml) for 24 hr (Fig 10B) could reduce activin βA, activin βB and follistatin mRNA levels in a time- and dose-dependent manner. In parallel experiments, the inhibitory effects of hCG on these gene targets could be mimicked by similar treatment with increasing doses (3–30 IU/ml) of equine LH (Fig 11A) but not FSH (Fig 11B). Although FSH treatment did not alter transcript expression of activin βA and follistatin, interestingly, activin βB mRNA was found to be up-regulated in a concentration-related fashion, implying that FSH may serve as a stimulator for activin βB expression in the carp pituitary. In contrast to the inhibitory actions of hCG, removal of endogenous LH by immunoneutralization using antiserum raised against carp LH was effective in elevating activin βA, activin βB and follistatin mRNA expression in carp pituitary cells (Fig 11C). To further examine the possible interactions between LH and GH in regulating activin/follistatin system, GH-induced activin β and follistatin gene expression were also tested with simultaneous exposure to hCG. In this case, the elevations in activin βA, activin βB and follistatin mRNA levels caused by GH induction (300 ng/ml) could be totally abolished by co-treatment with hCG (40 IU.ml, Fig 12A). In mammals, feedback regulation of activin/follistatin system at pituitary level via local production of follistatin induced by activin has been reported [51]). In carp pituitary cells, activin A (30 ng/ml) and B treatment (30 ng/ml) did not alter basal expression of activin βA and βB transcripts (data not shown) but consistently elevated follistatin mRNA levels (Fig 12B). In these experiments, co-treatment with hCG (40 IU/ml) not only could suppress basal but also block the stimulatory effects of activin A and B on follistatin gene expression.

## Discussion

Activin, a dimeric protein of TGFβ family composed of two β subunits, is known to have pleiotropic functions in different tissues/organs (see Introduction for details). To date, five isoforms of activin β subunits, namely βA, βB, βC, βD and βE, have been reported. Except for activin βD, which was cloned in *Xenopus* [52], the other forms of β subunits, including βA, βB, βC and βE, are all expressed in mammals with βA and βB as the dominant forms (for review, see [53]). Homodimerization of βA or βB, especially in tissues with a single form of activin β expression, can lead to the formation of activin A and activin B, respectively, while heterodimerization of βA and βB is known to form activin AB in tissues with βA and βB co-expression. Unlike βA and βB subunits, which are widely expressed at tissue level, βC and βE homo/heterodimers can be found only in the liver [54] and may play a role in apoptosis of cancer cells at the hepatic level [55]. As a first step to examine the functional role of pituitary activin/ follistatin system in GH regulation in carp species, grass carp activin βA and βB were cloned and shown to be single-copy genes in carp genome by Southern blot. Phylogenetic analysis also revealed that the newly cloned cDNAs of carp origin could be clustered into the respective clades of activin βA and βB in fish models and closely related to the corresponding sequences in goldfish and zebrafish, indicating that they are orthologues of the respective genes in grass carp. In agreement with this idea, the deduced protein sequences of carp activin βA and βB were found to be highly homologous to the corresponding sequences in other species, especially in the region for activin β mature peptide (75%–99% for βA and 93%–99% for βB subunit from fish to mammals). Within the mature peptide, the TGFβ signature motif as well as the nine cysteine residues for intra/intermolecular disulfide bonding, which contribute to the formation of a rigid eight-membered ring called “cysteine knot” in members of TGFβ family [56], were shown to be well conserved in the carp sequences. Based on 3D protein modelling, except for the minor changes in α2 and α3 helical domains, the spatial arrangement of the two pairs of antiparallel β strands containing the “cysteine knot”, which form the main core of β subunit for activin dimerization and receptor binding [57], were found to be highly comparable if not identical to their human counterparts. Our findings, taken together, provide evidence that the molecular structures of activin βA and βB are highly conserved in carp model.

At cellular level, activin can exert its effects by transactivation of activin-responsive elements (e.g., SMAD binding sites) in its target gene promoter [13] and activin actions in general can be nullified by its binding protein follistatiin, probably by preventing its binding to activin receptor [58]. Based on the βA and βB sequences obtained, recombinant grass carp activin A and B were expressed. In GH3 cells, these protein products were shown to be effective in stimulating promoter activation mediated by the pAR3-Lux reporter with 5’promoter carrying tandem repeats of activin-responsive elements originated from the human Mix2 promoter [44]. In the same study, the stimulatory effects of carp activin A and B were also negated by co-treatment with human follistatin. These findings indicate that the newly cloned cDNAs indeed encode functional proteins of carp origin with the ability to induce transactivation of target promoter with activin-responsive elements. The blockade by follistatin also suggest that human follistatin can bind with carp activin A and B to neutralize their bioactivity, which is in agreement with the high level of structural similarity observed between carp activin βs and their human counterparts. In mammal, activin was first isolated from the follicular fluid in the ovary by its stimulatory action on FSH release [1, 54]. Subsequent studies (e.g., in rodents) also reveal that it is widely expressed in non-ovarian tissues including the pituitary, placenta, liver, bone marrow, kidney, adrenal gland, skin and different brain areas [25, 59, 60], which is consistent with the pleiotropic functions reported for activin (see Introduction) and corroborates with the common findings that activin mainly serves as an autocrine/ paracrine factor rather than endocrine hormone [51]. Different isoforms of activin β subunit have also been cloned in fish models, e.g., in goldfish [29, 61], zebrafish [62] and rainbow trout [63], and tissue expression profiling in these studies consistently reveals that they are widely expressed at tissue level similar to that of mammals. The same is also true for our study in grass carp. As revealed by RT-PCR, except for the lack of βA signal in muscle and spinal cord, activin βA and βB were found to be expressed in all the tissues and brain areas examined in carp model. In our study, high levels of βA and βB signals were located in the brain (especially in the telencephalon, optic tectum and hypothalamus) as well as in the gut, liver and kidney, although lower levels of βA and βB signals could also be noted in the pituitary and other tissues. Interestingly, activin βB was shown to be the major signal (or even the only signal) detected in the heart, gill, muscle, spleen and spinal cord, whereas activin βA was found to the dominant form expressed in the liver. The differential expression of βA and βB subunits may serve as an intrinsic mechanism to modulate the relative proportion of the protein products including activin A, activin B and activin AB at tissue level, although the biological functions of the three forms of activin have yet to be examined in carp model.

In mammals (e.g., in rat), cell type-specific expression of different components of actvin/follistatin system has been reported in the pituitary, with activin βB expressed in gonadotrophs [16] and activin βA and follistatin expressed in folliculostellate cells [64]. Activin signals have also been demonstrated in human gonadotroph adenomas [47], suggesting a possible link between activin and carcinogenesis at pituitary level [14]. To our knowledge, there is only a single report in goldfish on cell type-specific expression in the pituitary of non-mammalian species. In this case, immunoreactivity of activin βB but not βA was found in goldfish somatotrophs and none of the activin signals (βA & βB) could be located in gonadotrophs [34], implying that the “spatial arrangement” of the pituitary activin/ follistatin system may be different between fish and mammals. In our study, transcript expression of activin βA, activin βB, follistatin and activin receptors including ActRIB and ActRIIB were detected in grass carp pituitary cells, indicating that a functional activin/follistation system may be present in the carp pituitary. Using RT-PCR coupled with LCM isolation of immuno-identified pituitary cells, ActRIB and ActRIIB were confirmed to be expressed in carp lactotrophs, gonadotrophs and somatotrophs while the corresponding signals for activin βB could be noted mainly in carp gonadotrophs, to a lesser extent in lactotrophs, but not in somatotrophs. In contrast, activin βA was not expressed in the three cell types examined, despite its signal was detected in mixed populations of pituitary cells, implying that activin βA is expressed in cell types other than lactotrophs, gonadotrophs and somatotrophs. Our finding of activin βB expression in gonadotrophs is at variance with the study in goldfish [34] but is more comparable with the rat model [16]. In our study, co-expression of ligand (activin βB) with its receptors (ActRIB & ActRIIB) in the same cell type (lactotrophs & gonadotrophs) as well as expression of activin receptors in neighbouring cells (somatotrophs) may form the basis for autocrine/paracrine regulation of pituitary hormones by activin in carp species. Given that (i) only the transcript signal for activin βB was detected in the carp pituitary by Northern blot, and (ii) activin βA was not expressed in carp lactotrophs, gonadotrophs and somatotrophs, which constitute ~82% of the cell populations within the anterior pituitary of grass carp [35], it would be logical to assume that activin βB is the major form of β subunit expressed at pituitary level in carp model. Of note, βA and βB co-expression was not detected in the three cell types examined (i.e., no activin AB formation) and the highest level of βB subunit expression could be found in carp gonadotrophs. It is likely that activin B produced by gonadotrophs (and to a less extent by lactotrophs) may serve as a major source of activin in the carp pituitary.

Based on extensive studies in rodents and other species, activin is known to be a key component for reproductive functions [10] and activin levels in circulation have been proposed to be a “predictor” for reproductive health in human female [65]. At pituitary level, activin is well-documented for its ability to induce FSH synthesis and secretion [17] and its stimulatory effect on FSHβ gene transcription is mediated by SMAD3 and SMAD4 [19] and their synergistic interactions with AP-1 [66] and FOXL2 transcription factors [18]. Although GH regulation by activin has also been reported, the effects are controversial with both stimulatory [23, 24] and inhibitory actions reported for GH secretion and GH gene expression [25–27]. Unlike FSH studies, the transcriptional mechanisms, especially the role of SMADs, for GH regulation by activin are still unclear and remain to be characterized. In fish models, only three papers have been published to date for the pituitary actions of activin on GH regulation, with one in goldfish showing a stimulatory effect on GH secretion [34] and the other in zebrafish showing an attenuation in GH mRNA level [28]. In a recent report in European eel, however, activin was found to have no effect on GH gene expression [30]. To shed light on the functional role and underlying mechanisms for GH regulation by activin in grass carp, a systematic study was conducted in primary culture of carp pituitary cells. In this case, static incubation with activin A and B was shown to reduce GH release and GH cell content with a parallel drop in GH mRNA levels. These parameters indicative of GH synthesis and secretion, however, could be up-regulated by removing endogenous activins with follistatin, implying that activin produced locally at the pituitary level may act as an autocrine/paracrine signal to inhibit GH expression and secretion in carp model. In carp pituitary, GH [36] and LH released locally [40] are known to induce GH gene expression and constitute an “intrapituitary feedback loop” for GH regulation [39]. In our study, activin A and B not only could suppress basal but also block the stimulatory effects of GH and hCG on GH mRNA expression. These findings also raise the possibility that activin may be involved in GH regulation mediated by local actions of GH and LH in carp pituitary cells.

Regarding the mechanisms for the inhibitory effect of activin on GH gene expression, activin A and B were both effective in reducing GH primary transcript levels with notable enhancement in GH mRNA clearance (as reflected by a decrease in *T*_*1/2*_ of GH transcript) in carp pituitary cells, suggesting that both transcriptional (by reducing GH gene transcription) and post-transcriptional mechanisms (by decreasing GH transcript stability) are involved in the drop of GH mRNA levels observed after activin treatment. In mammals, GH regulation by altering GH transcript stability has been reported, e.g., up-regulation of GH gene expression by glucocorticoid and thyroid hormone via an enhancement in GH mRNA stability by lengthening its 3’poly(A^+^) tail [67]. However, a functional link between GH transcript stability and activin treatment has not been reported previously. For transcriptional regulation of GH gene in grass carp, our idea is also supported by parallel studies with GH3 cells, a somatotroph cell line responsive to activin induction [24]. In this cell model, promoter activity conferred by a 986 bp grass carp GH promoter could be attenuated in a dose-dependent manner by activin A and B treatment. By 5’deletion analysis, the activin-responsive region was mapped to the sequence covering position -742 to -656 of GH promoter. At cellular level, the biological actions of activin are known to be mediated by the SMAD pathway functionally coupled with activin receptors, especially through SMAD2 and SMAD3 [11, 13]. Within the 986 bp GH promoter of carp origin [68], eight putative SMAD binding sites could be found and two of them (GTCT & TCTG) were located in the region between -742 to -656, suggesting that the SMAD pathway may be involved in GH regulation by activin in carp model. Given that (i) SMAD2 and SMAD3 expression were both effective in mimicking the down-regulation of GH promoter activity by activin in GH3 cells, and (ii) the inhibitory effects of activin A and B on GH promoter activation in the same cell model could be reverted by expression of DN mutants of SMAD2 and SMAD3, it would be logical to assume that activin could inhibit GH gene transcription in carp pituitary cells by reducing GH promoter activity via SMAD-dependent mechanisms.

Although activin is known to regulate pituitary hormone secretion/gene expression, including FSH [17], LH [22], PRL [24] and GH [23, 25], reciprocal modulation of activin by pituitary hormones has also been reported. For examples, FSH and LH are both effective in stimulating activin βA and βB gene expression in granulosa cells/granulosa-luteal cells of rat [69] and human origin [70]. In zebrafish follicular cells, interestingly, hCG treatment can lead to differential effects on the two β subunits, with stimulation on βA but inhibition on βB mRNA expression [71]. To our knowledge, activin regulation by GH has not been examined and it is still unclear if the activin/follistatin system can also serve as a regulatory target for GH at the pituitary level. In our studies with carp pituitary cells, activin βA, activin βB and follistain mRNA expression could be up-regulated by GH treatment but the opposite effects with inhibitory actions on these gene targets were noted by removing endogenous GH with immuno-neutralization using GH antiserum, suggesting that GH released locally may play a role in maintaining/ enhancing the activin/follistatin system at the pituitary level. Using a pharmacological approach, GH-induced activin βA and βB mRNA expression were also found to be sensitive to the blockade of JAK_2_ (by AG490), STAT_5_ (by IQDMA), MEK_1/2_ (by U0126), ERK_1/2_ (by FR180204), PI3K (by LY294002) and Akt (by HIMOC), indicating that the activin responses induced by GH were mediated via JAK_2_/ STAT_5_, MEK_1/2_/ERK_1/2_ and PI3K/Akt cascades. In mammals, functional coupling of JAK_2_/STATs, MAPK and PI3K/Akt pathways with GH receptor has been well documented [50] and similar findings have also been reported in fish models, e.g., in rainbow trout [72] and grass carp [73]. In carp model, local release of GH is known to induce GH synthesis and secretion via autocrine stimulation of carp somatotrophs [36], which constitutes a key component of the “intrapituitary feedback loop” formed by local interactions of LH and GH [39]. Our findings of (i) GH induction of activin/follistatin expression and (ii) activin inhibition of GH synthesis and secretion in carp pituitary cells also suggest that pituitary expression of activin may serve as a negative feedback to tune down the GH responses induced by GH autoregulation via autocrine mechanisms.

In the carp pituitary, local release of LH may also play a role in autocrine/paracrine regulation of the activin/follistatin system. This idea is supported by our findings that hCG could suppress activin βA, activin βB and follistain mRNA expression in carp pituitary cells in a time- and dose-dependent manner and these inhibitory effects were mimicked by similar treatment with LH but not FSH. However, the opposite was true with parallel rises of these gene targets after removing endogenous LH with immuno-neutralization using LH antiserum, indicating that local release of LH by activating LH receptor but not FSH receptor can serve as a negative regulator of the activin/follistatin system in the carp pituitary. In mammals, activin-induced follistatin expression to nullify activin’s actions is well documented at tissue level [58] and a similar local feedback for activin/follistatin system appears to be well conserved in fish models, e.g., in zebrafish [74] and goldfish [31]. In our studies, follistatin mRNA expression could be notably enhanced by activin A and B in carp pituitary cells and these stimulatory actions were negated by hCG co-treatment. Besides the effect on activin-induced follistatin gene expression, hCG was also effective in blocking GH-induced activin βA, activin βB and follistain mRNA expression at the pituitary level. These findings, as a whole, suggest that local release of LH not only can inhibit activin feedback via follistatin production but also suppress GH modulation of activin/follistatin system in carp pituitary. Given that follistatin expression induced by activin could be abrogated by hCG treatment, the inhibitory effect of LH on follistatin gene expression is likely the result of a direct action rather than an indirect effect through the drop in activin expression induced by LH in carp pituitary cells.

**Fig 13:**
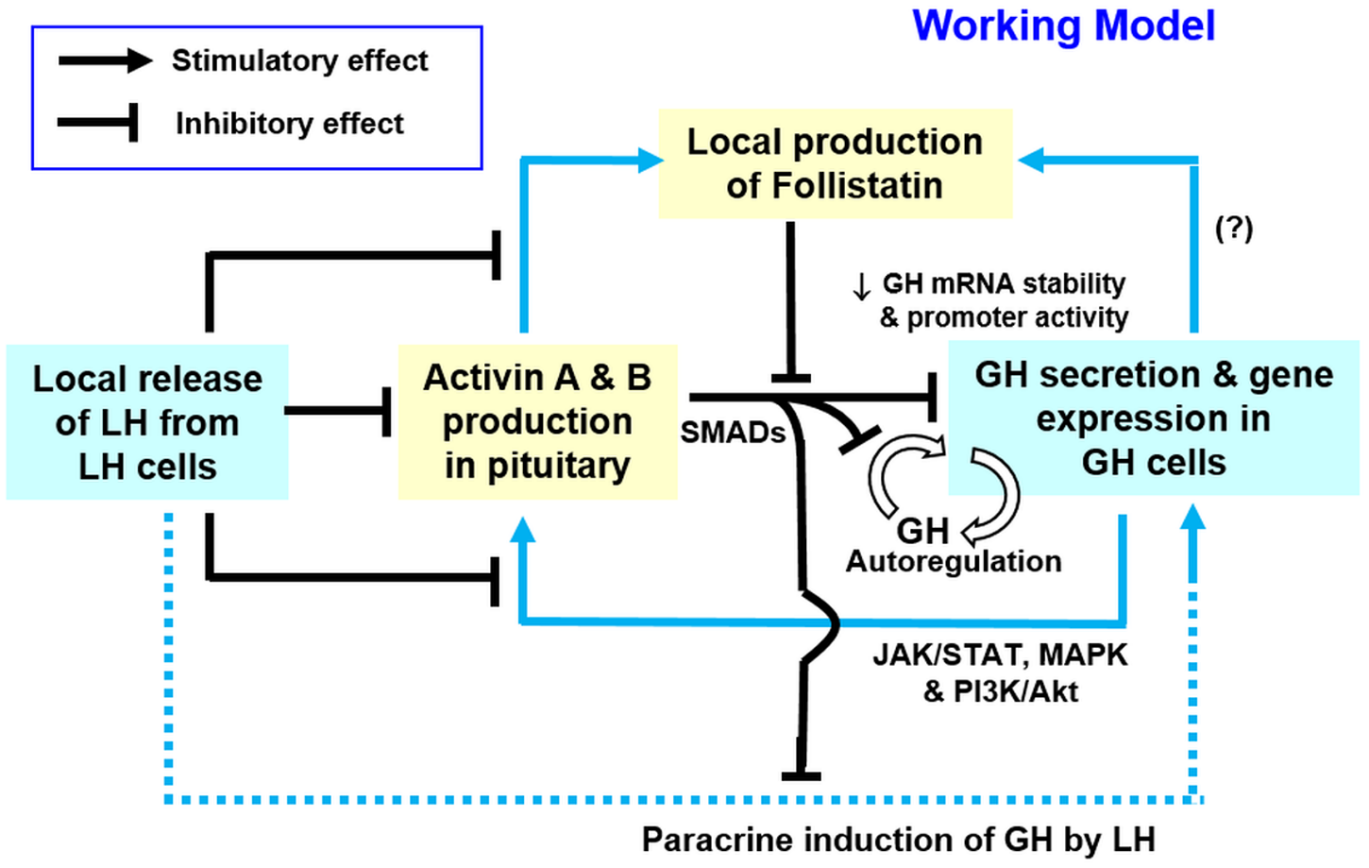
Functional interactions of GH and LH with activin/follistatin system in the carp pituitary. In carp pituitary, local release of GH not only can induce GH release and gene expression via GH autoregulation in somatotrophs (GH cells) but also up-regulate the pituitary activin/follistatin system via JAK_2_/STAT_5_, MEK_1/2_/ERK_1/2_ and PI3K/Akt cascades. Activin A and B production induced by GH can exert a negative feedback to suppress GH synthesis and secretion and the inhibitory effect on GH gene expression is mediated by a drop in GH mRNA stability with parallel inhibition on GH promoter activity via SMAD2/3. Beside the effects on GH, activin A and B can also inhibit the paracrine induction of LH on GH gene expression. Meanwhile, the local effects of activin A and B could be blocked by activin-induced follistatin expression at pituitary level, which forms an intrinsic feedback for the activin/follistatin system. Furthermore, activin-induced follistatin expression as well as GH up-regulation of activin/follistatin system is also under the negative regulation of LH released from gonadotrophs (LH cells). The activin/follistatin system with functional interactions with GH and LH released locally at the pituitary level constitutes a new facet for autocrine/ paracrine regulation of GH expression in carp species.

In summary, grass carp activin βA and βB have been cloned, their gene copy number and expression profile at tissue level, especially in the pituitary, have been characterized, and the functionality of their protein products, namely activin A and B, has been confirmed by their ability to induce transactivation of target promoter with activin-responsive elements. Based on our studies with carp pituitary cells and GH3 cells, a working model for the functional role of activin/follistatin system in GH regulation by GH and LH released locally at the pituitary level has been proposed for carp species (Fig 13). In this model, local release of GH via paracrine actions can stimulate activin A and B expression in the carp pituitary via JAK_2_/STAT_5_, MAPK and PI3K/Akt pathways. Activin A and B produced in turn exert a negative feedback to inhibit GH synthesis and secretion with parallel drop in GH mRNA in carp somatotrophs. Activin A and B not only suppress basal but also negate the stimulatory effects of GH (via GH auto-regulation) and LH (by paracrine induction) on GH gene expression. Apparently, the inhibitory effect of activins on GH gene expression is mediated through a drop in GH transcript stability and SMAD2/3 inhibition on GH promoter activity. Of note, activin A and B can also induce follistatin expression at pituitary level, which constitutes a local feedback for activin’s actions on GH expression. Interestingly, local release of LH may form another level of negative signals to inhibit activin induction of follistatin expression as well as GH modulation of activin/follisatin system in the carp pituitary. At present, it is still not sure if the effect of GH on follistatin expression is mediated by a direct stimulation of GH or via indirect action by its stimulation on activin production, although the two are not mutually exclusive. Our findings, as a whole, provide evidence that the intrapituitary activin/follistatin system can serve as a regulatory target for local interactions of GH and LH and contribute to GH regulation by autocrine/ paracrine mechanisms in the carp pituitary. Given that the involvement of SMAD pathway in activin’s action has also been reported in fish model, e.g., in FSHβ expression in goldfish [75], studies are now underway in our laboratory to examine the role of SMAD binding sites identified in the grass carp GH promoter in the regulatory action of activin on GH gene transcription.

## Supporting information

S1 Fig Nucleotide and amino acid (a.a) sequences of grass carp activin βA subunit. The full-length cDNA of carp activin βA contains a 1212 bp ORF encoding a 404 a.a. activin βA precursor. The ORF region is presented in upper case while the 5’UTR and 3’UTR are presented in lower case. The signal peptide is marked with a dotted underline and the mature peptide for carp activin βA is underlined with a black solid line. The N-linked glycosylation site (N-glycosylation site) and protein cleavage site preceding the mature peptide are boxed by dotted line and solid line, respectively. The nine conserved cysteine residues located within the mature peptide (for disulfide bonding) are shaded in grey and the polyadenylation site identified in 3’UTR is underlined in italic for recognition.
(TIF)

S2 Fig Nucleotide and amino acid (a.a) sequences of grass carp activin βB subunit. The full-length cDNA of carp activin βB contains a 1176 bp ORF encoding a 392 a.a. activin βB precursor. The ORF region is presented in upper cases while the 5’UTR and 3’UTR are presented in lower cases. The signal peptide is marked with a dotted underline and the mature peptide for carp activin βB is underlined with a black solid line. The N-linked glycosylation site (N-glycosylation site) and protein cleavage site preceding the mature peptide are boxed by dotted line and solid line, respectively. The nine conserved cysteine residues located within the mature peptide (for disulfide bonding) are shaded in grey and the four polyadenylation sites identified in 3’UTR is underlined in italic for recognition.
(TIF)

S3 Fig Protein sequence alignment of grass carp activin βA with the corresponding sequences reported in other vertebrates. The sequence alignment was conducted using Clustal-W algorithm. The conserved residues within the protein sequences are boxed in gray. The signal peptide is marked by dotted underline while the mature peptide for activin βA is underlined with a black solid line. The signature motif for TGFβ family located within the activin mature peptide is boxed with red line and the nine conserved cysteine residues (for disulfide bonding) are marked by inverted arrows.
(TIF)

S4 Fig Protein sequence alignment of grass carp activin βB with corresponding sequences reported in other vertebrates. The sequence alignment was conducted using Clustal-W algorithm. The conserved residues within the protein sequences are boxed in gray. The signal peptide is marked by dotted underline while the mature peptide for activin βB is underlined with a black solid line. The signature motif for TGFβ family located within the activin mature peptide is boxed with red line and the nine conserved cysteine residues (for disulfide bonding) are marked by inverted arrows.
(TIF)

## Abbreviations

Akt: Protein kinase B
ERK_1/2_: Extracellular signal-regulated kinase ½
FSH: Follicle stimulating hormone
GH: Growth hormone
hCG: Human chorionic gonadotropin
JAK_2_: Janus kinase 2
LH: Luteinizing hormone
MAPK: Mitogen-activated protein kinases
MEK_1/2_: Mitogen-activated protein kinase kinase 1/2
PI3K: Phosphoinositide 3-kinase
STAT_5_: Signal transducer and activator of transcription 5
