# Supplementary figures and images for: Activin/follistatin system in grass carp pituitary cells: - Regulation by local release of growth hormone and luteinizing hormone and its functional role in growth hormone synthesis and secretion
Source: PLoS One. 2017 Jun 29;12(6):e0179789. doi: 10.1371/journal.pone.0179789 (PMC5491050; doi:10.1371/journal.pone.0179789)

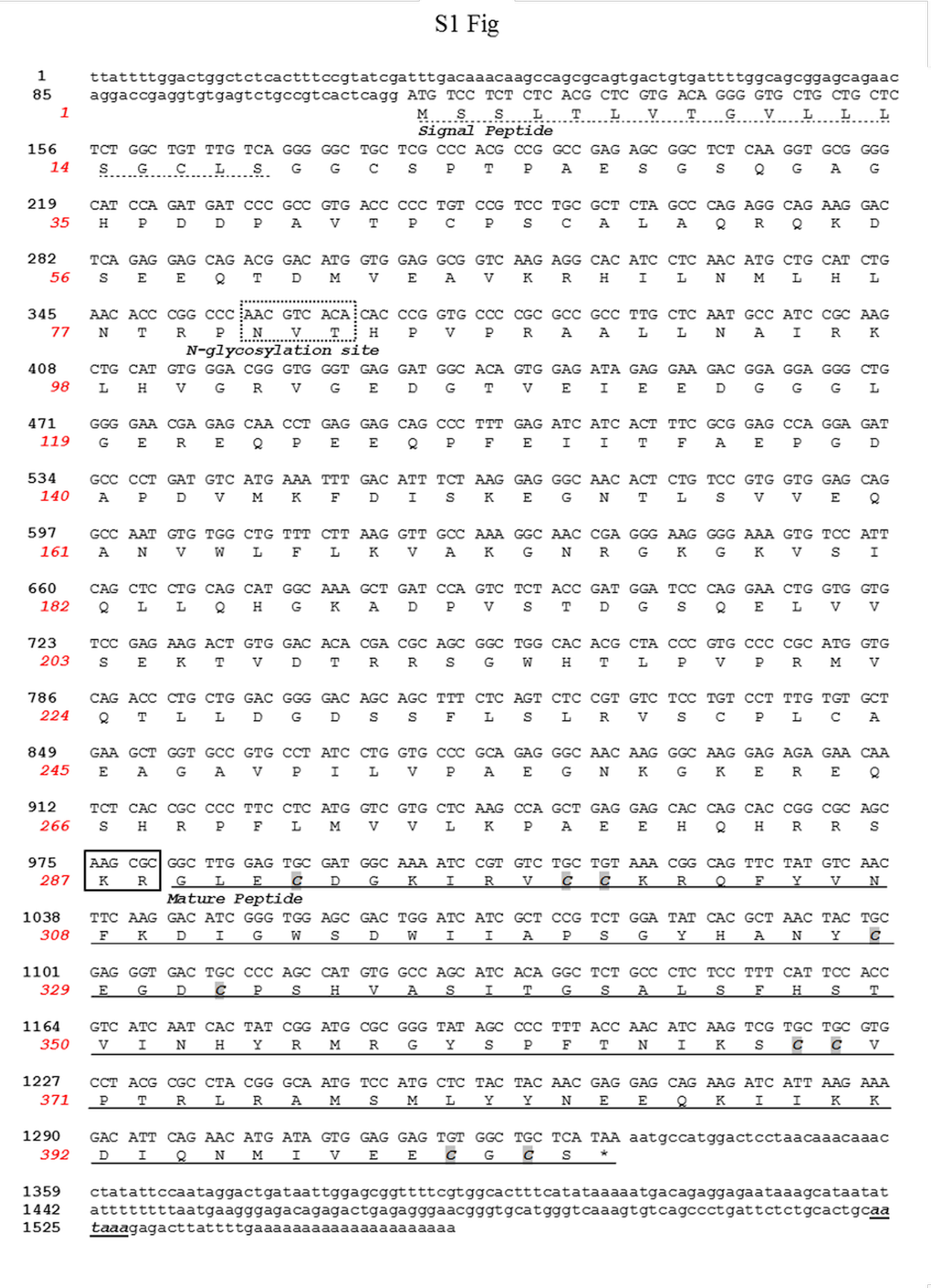

Supplement: S1 Fig — The full-length cDNA of carp activin βA contains a 1212 bp ORF encoding a 404 a.a. activin βA precursor. The ORF region is presented in upper case while the 5’UTR and 3’UTR are presented in lower case. The signal peptide is marked with a dotted underline and the mature peptide for carp activin βA is underlined with a black solid line. The N-linked glycosylation site (N-glycosylation site) and protein cleavage site preceding the mature peptide are boxed by dotted line and solid line, respectively. The nine conserved cysteine residues located within the mature peptide (for disulfide bonding) are shaded in grey and the polyadenylation site identified in 3’UTR is underlined in italic for recognition. (TIF) [file pone.0179789.s001.tif]

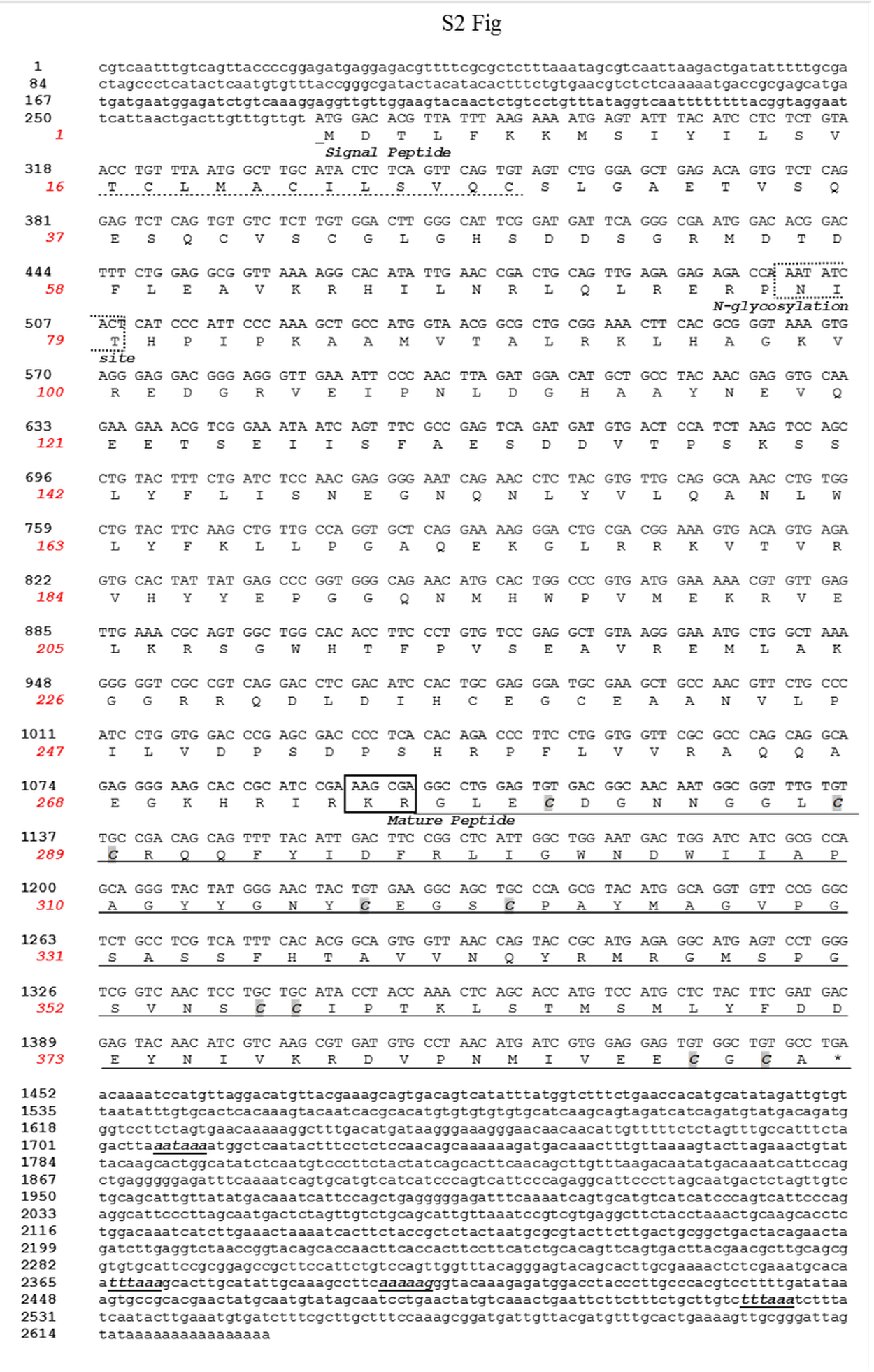

Supplement: S2 Fig — The full-length cDNA of carp activin βB contains a 1176 bp ORF encoding a 392 a.a. activin βB precursor. The ORF region is presented in upper cases while the 5’UTR and 3’UTR are presented in lower cases. The signal peptide is marked with a dotted underline and the mature peptide for carp activin βB is underlined with a black solid line. The N-linked glycosylation site (N-glycosylation site) and protein cleavage site preceding the mature peptide are boxed by dotted line and solid line, respectively. The nine conserved cysteine residues located within the mature peptide (for disulfide bonding) are shaded in grey and the four polyadenylation sites identified in 3’UTR is underlined in italic for recognition. (TIF) [file pone.0179789.s002.tif]

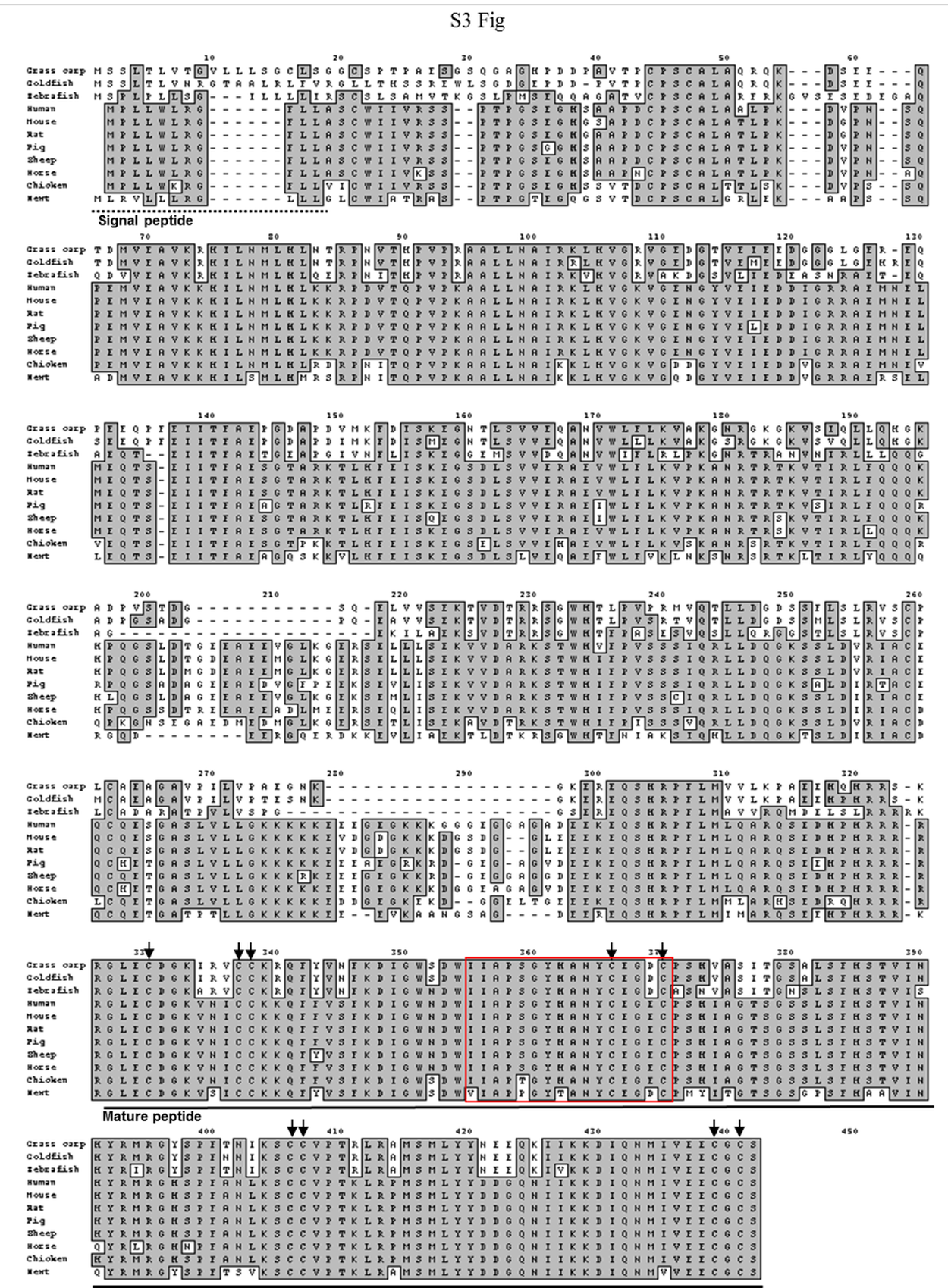

Supplement: S3 Fig — The sequence alignment was conducted using Clustal-W algorithm. The conserved residues within the protein sequences are boxed in gray. The signal peptide is marked by dotted underline while the mature peptide for activin βA is underlined with a black solid line. The signature motif for TGFβ family located within the activin mature peptide is boxed with red line and the nine conserved cysteine residues (for disulfide bonding) are marked by inverted arrows. (TIF) [file pone.0179789.s003.tif]

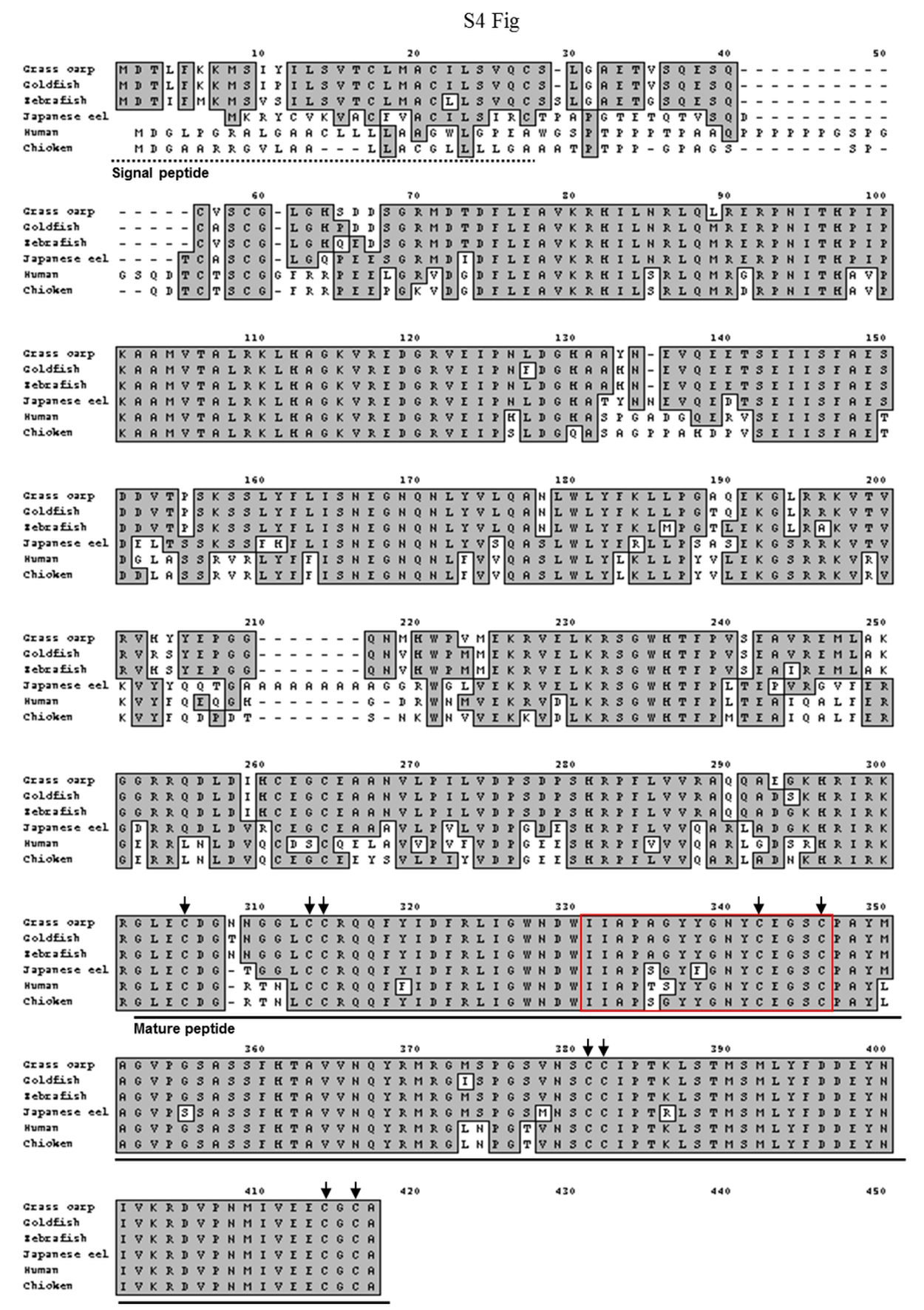

Supplement: S4 Fig — The sequence alignment was conducted using Clustal-W algorithm. The conserved residues within the protein sequences are boxed in gray. The signal peptide is marked by dotted underline while the mature peptide for activin βB is underlined with a black solid line. The signature motif for TGFβ family located within the activin mature peptide is boxed with red line and the nine conserved cysteine residues (for disulfide bonding) are marked by inverted arrows. (TIF) [file pone.0179789.s004.tif]
